# Genome-wide identification, phylogeny, and expression analysis of the SBP-box gene family in Euphorbiaceae

**DOI:** 10.1186/s12864-019-6319-4

**Published:** 2019-12-24

**Authors:** Jing Li, Xiaoyang Gao, Shiye Sang, Changning Liu

**Affiliations:** 1 CAS Key Laboratory of Tropical Plant Resources and Sustainable Use, Xishuangbanna Tropical Botanical Garden, Chinese Academy of Sciences, Kunming, 650223 China; 2 College of Life Sciences, University of Chinese Academy of Sciences, Beijing, 100049 China; 3 Center of Economic Botany, Core Botanical Gardens, Chinese Academy of Sciences, Menglun, Mengla, 666303 Yunnan China

**Keywords:** Euphorbiaceae, SBP-box, *miR156*, Tissue expression, Stress response, Gene duplication

## Abstract

**Background:**

Euphorbiaceae is one of the largest families of flowering plants. Due to its exceptional growth form diversity and near-cosmopolitan distribution, it has attracted much interest since ancient times. SBP-box (*SBP*) genes encode plant-specific transcription factors that play critical roles in numerous biological processes, especially flower development. We performed genome-wide identification and characterization of *SBP* genes from four economically important Euphorbiaceae species.

**Results:**

In total, 77 *SBP* genes were identified in four Euphorbiaceae genomes. The SBP proteins were divided into three length ranges and 10 groups. Group-6 was absent in *Arabidopsis thaliana* but conserved in Euphorbiaceae. Segmental duplication played the most important role in the expansion processes of Euphorbiaceae *SBP* genes, and all the duplicated genes were subjected to purify selection. In addition, about two-thirds of the Euphorbiaceae *SBP* genes are potential targets of *miR156*, and some miR-regulated *SBP* genes exhibited high intensity expression and differential expression in different tissues. The expression profiles related to different stress treatments demonstrated broad involvement of Euphorbiaceae *SBP* genes in response to various abiotic factors and hormonal treatments.

**Conclusions:**

In this study, 77 *SBP* genes were identified in four Euphorbiaceae species, and their phylogenetic relationships, protein physicochemical characteristics, duplication, tissue and stress response expression, and potential roles in Euphorbiaceae development were studied. This study lays a foundation for further studies of Euphorbiaceae *SBP* genes, providing valuable information for future functional exploration of Euphorbiaceae *SBP* genes.

## Background

Transcription factors (TFs) are DNA-binding proteins that play essential roles in the regulatory networks of critical developmental processes [1]. According to the specific protein structure, TFs can be divided into distinct families. *SQUAMOSA* promoter-binding protein (SBP)-box (briefly: *SBP*) or SBP-like (*SPL*) genes encode a type of TF family that is uniquely conserved in plants. *SBP* genes were first identified in *Antirrhinum majus*, and they were found to regulate the expression of MADS-box genes, which are critical in floral development [2]. Since then, studies on *SBP* genes have continually been carried out. As a result, *SBP* genes have continually been identified in plants ranging from monocyte algae to flowering plants [3, 4]. It has been reported that *SBP* genes play critical roles in regulating flowering, fruit ripening, phase transition, and other physiological processes. In *Arabidopsis thaliana*, *AtSPL3, AtSPL4*, and *AtSPL5* are direct upstream activators of LEAFY, FRUITFULL, and APETALA1, and they redundantly promote flowering [5]. They also integrate developmental aging and photoperiodic signals in a process that involves the flowering locus T (FT)-flowering locus D (FD) module in *A. thaliana* [6]. In addition, *AtSPL9* and *AtSPL15* as well as *AtSPL2, AtSPL10*, and *AtSPL11* are regarded as regulators of plastochron and branching [7, 8]. *AtSPL1* and *AtSPL12* have been reported to play roles in plant thermotolerance during the reproductive stage [9]. *AtSPL7* is a regulator of copper homeostasis and responses to light and copper [10]. There are also reports on *SBP* genes of other species: an *SBP* gene in *Solanum lycopersicum* (tomato) is critical for normal ripening [11]; *OsSPL16* of *Oryza sativa* (rice) is a regulator of grain size, shape, and quality [12]; and *OsSPL14* plays a role in controlling tiller growth in rice [13].

*SBP* genes encode a class of proteins that have a conserved DNA-binding domain (SBP-specific domain) that contains about 75 amino acid residues (aa). The SBP-specific domain is sufficient to bind to the GTAC core motif [2, 14–16]. There are three common structures in all SBP-specific domains: two zinc fingers and a nuclear localization signal (NLS). The NLS and the second zinc finger partly overlap [16]. Additionally, some *SBP* genes can be regulated by miRNAs (about 22–24 nt), which reduce protein levels at the transcriptional or translational stage by complementarily binding to their target mRNAs [17–19]. *MiR156* plays the most important regulatory roles out of almost all the miRNAs that regulate *SBP* genes (with target sites located either in the coding region [CDS] or 3′ untranslated region [UTR]) [20, 21]. It has been predicted that 10 of the 16 *AtSPL* genes are potential targets of *miR156/157* (collectively known as *miR156*). Due to regulation by miRNAs, some *SBP* genes are involved in complex regulatory processes. For example, *miR156* improves the drought tolerance of *Medicago sativa* by silencing *SPL13* [22] and it regulates the juvenile-to-adult phase transition by regulating downstream target *SBP* genes [5, 6, 23]. Additionally, via *miR156* regulation, *AtSPL3* temporally regulates shoot development in *A. thaliana* [24].

Euphorbiaceae is a large and widespread plant family that consists of more than 8000 species, including herbs, perennial shrubs, and trees. They are evolutionarily diverse, and have various traits that allow them to adapt to dynamic environmental conditions. With the increasing demand for food, industrial raw materials, ornamental plants, and herbal medicines, Euphorbiaceae plants have become increasingly attractive. There are many agri-economically important Euphorbiaceae species that have been widely cultivated, such as *Ricinus communis* (castor bean), *Manihot esculenta* (cassava), *Jatropha curcas* (physic nut), and *Hevea brasiliensis* (rubber tree). Castor bean can be cultivated at a large range of latitudes, and its oil is an important industrial raw material for producing lubricants and paints [25, 26]. Cassava has a starch-enriched root, and it has been a crucial food crop and is also ideal for bioethanol production [27, 28]. Physic nut has seeds with a high oil content that can be processed into biodiesel [29, 30]. The rubber tree is the most important source of natural rubber production, which is indispensable in daily life [31]. However, there are few studies on these non-model plants. More in-depth research, such as understanding the structure, evolution, and function of key gene families, is required to improve crop productivity and commercialization.

The SBP-box gene family has been identified and characterized in different plant species, such as *A. thaliana* [14], *Malus domesrica* (apple) [32], *Physcomitrella patens* (a moss species) [4], and *Zea mays* (maize) [33]. However, the *SBP* genes in Euphorbiaceae, and their evolutionary and functional characteristics, are rarely studied. Fortunately, the continuous publication of genome sequencing data [34–37] allows more in-depth research to be conducted on the Euphorbiaceae SBP-box gene family. Herein, we performed a genome-wide investigation of the SBP-box gene family in four Euphorbiaceae species. 77 *SBP* genes were identified using both local protein–protein Basic Local Alignment Search Tool (BLASTP) and hidden Markov model (HMM) searches. These genes were divided into three length ranges, and into 10 well-defined groups based on total sequence similarity and structural conservation. Duplication events and synteny blocks also supported our grouping scheme and revealed the details of the expansion process of Euphorbiaceae *SBP* genes. Additionally, a large amount of Euphorbiaceae *SBP* genes can be regulated by *miR156*. According to the expression profiles associated with different tissues and stress treatments, a large amount of miR-regulated *SBP* genes are highly differentially expressed in different tissues and the stress responses are ubiquitous among either miR-regulated or non-regulated *SBP* genes. Thus, we conducted a comprehensive analysis of Euphorbiaceae *SBP* genes, and provided valuable evolutionary information for further research.

## Results

### Identification and characterization

Previous studies on the SBP-box gene family have mainly focused on the model plant *A. thaliana*. There are few studies on non-model plants such as Euphorbiaceae plants. Zhang and Ling reported on the identification and structural analysis of castor bean *SBP* genes, but they provided little function prediction information [38]. Here, we performed a comparative analysis of *SBP* genes from four representative Euphorbiaceae species: cassava, rubber tree, physic nut, and castor bean (Table 1). We systematically identified and characterized the *SBP* genes of Euphorbiaceae, and predicted their potential functions.

**Table 1:** *SBP* gene members and data sources

Plant species	Common name	Gene number	Genome size (Mb)	References
*Arabidopsis thaliana*	Thale cress	16	115	[14]
*Manihot esculenta*	Cassava	21	562	This study
*Hevea brasiliensis*	Rubber tree	26	1290	This study
*Jatropha curcas*	Physic nut	15	308	This study
*Ricinus communis*	Castor bean	15	341	[38]

To comprehensively identify the *SBP* genes of each Euphorbiaceae species, we performed a whole-genome scan to identify protein-coding genes containing the SBP-specific domain by using both BLASTP and HMM search, and we then removed the proteins with incomplete SBP-specific domains. A total of 77 *SBP* genes containing 145 transcripts were identified (Additional file 1: Table S1). For each Euphorbiaceae species, the number of *SBP* genes varied from 15 to 26, comprising 15 in physic nut, 15 in castor bean, 21 in cassava, and 26 in rubber tree. The number of *SBP* genes was closely associated with genome size. For example, rubber tree and cassava had a relatively large number of *SBP* genes and they both experienced a recent genome duplication event [34, 39].

**Fig. 1:**
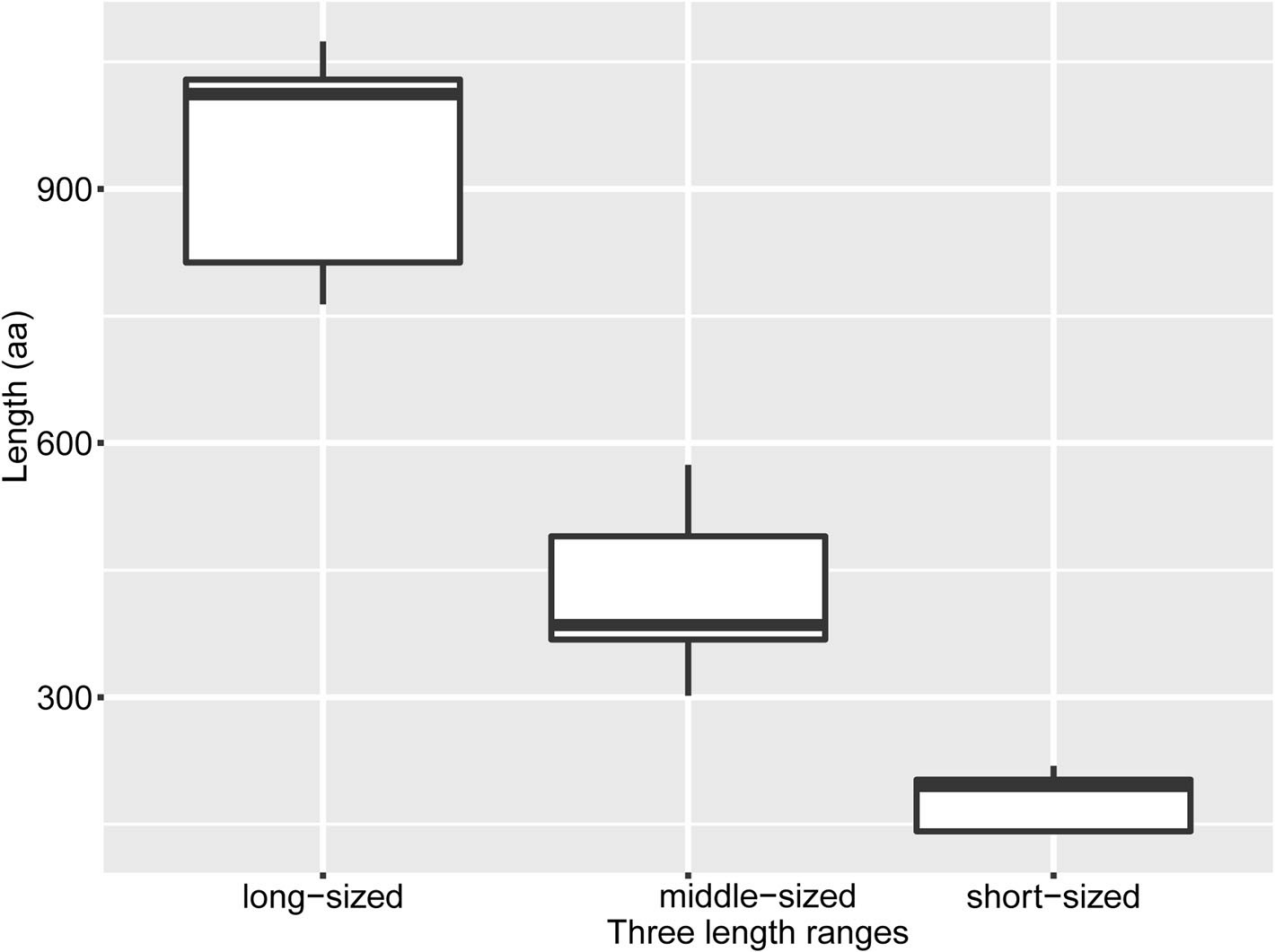
The distribution of three length ranges of SBPs. Y-axis represents protein length (aa); X-axis lists three length ranges

To further characterize the SBP proteins, the basic properties including protein length, isoelectric point value, and molecular weight were analyzed (Additional file 1: Table S2). The Euphorbiaceae SBPs covered a large range of lengths (140–1074 aa). Notably, the lengths exhibited a trimodal distribution (Fig. 1, Additional file 1: Table S2). The short-sized SBPs contained 140–219 aa with an average length of 182 aa; the middle-sized SBPs contained 302–557 aa with an average length of 418 aa; and the long-sized SBPs contained > 780 aa with an average length of 956 aa. The number of *SBP* genes in the short-, middle-, and long-sized length categories were: 15, 41, and 21, respectively. The corresponding molecular masses were 15.69–24.4, 33.94–63.49, and 85.6–119.32 kDa, respectively.

### Phylogenetic analysis and classification

**Fig. 2:**
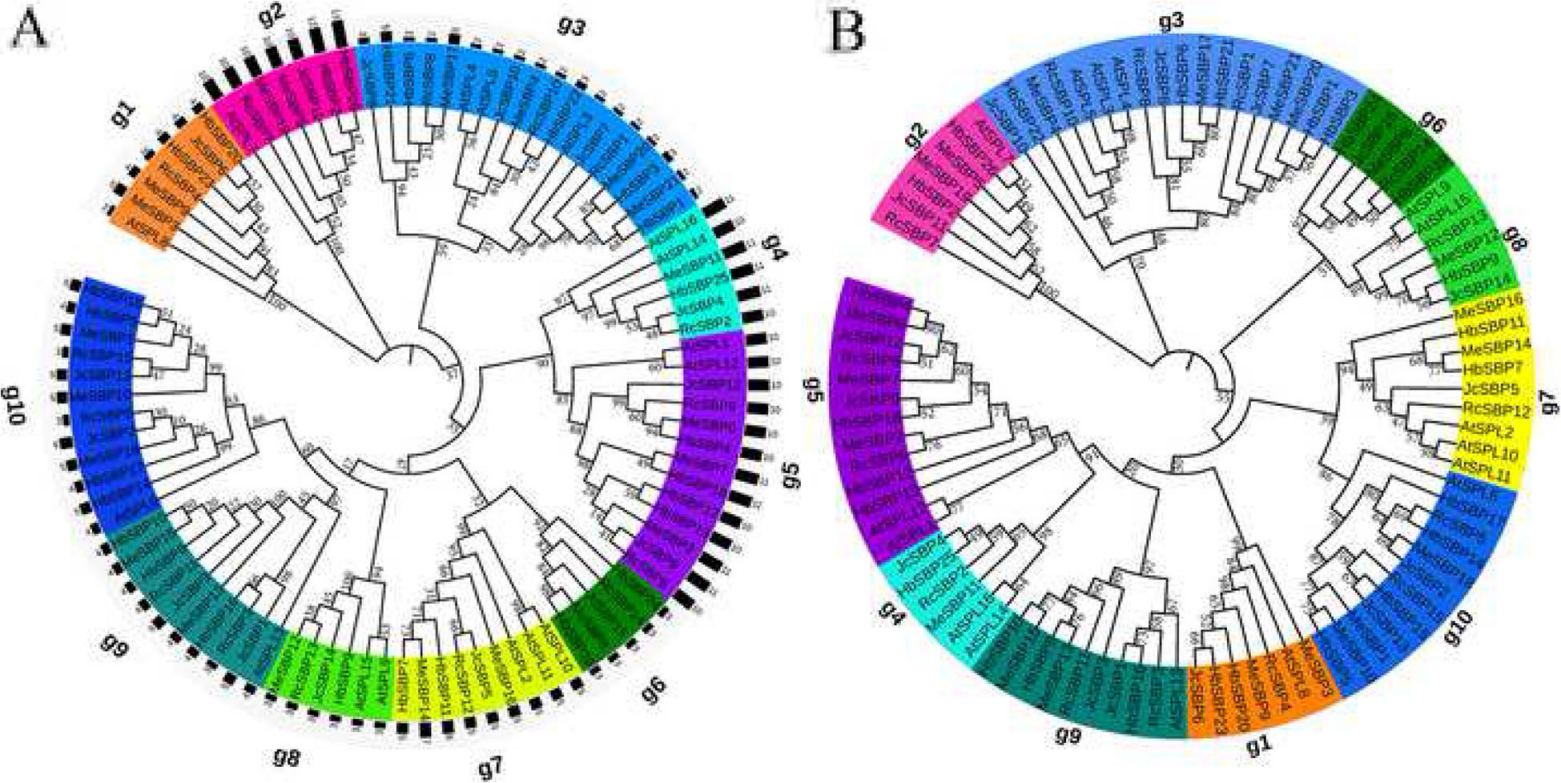
The phylogenetic tree. The neighbor-joining tree (**a**) was created using the MEGA7.0 program (bootstrap value set at 1000). The maximum likelihood tree (**b**) was constructed by PAUP* program. All these SBP proteins were divided into 10 groups, respectively are: *g1*, *g2*, *g3*, *g4*, *g5*, *g6*, *g7*, *g8*, *g9*, *g10*. The *SBP* genes in a specific group were marked with a specific color. The bootstrap values were marked by percentage, ‘%’ was omited. The intron number for each *SBP* gene was displayed in a black bar outmost (**a**)

To better understand the functions and evolutionary trajectory of the Euphorbiaceae *SBP* genes, a phylogenetic analysis of the 77 Euphorbiaceae SBPs plus 16 *A. thaliana* SPLs was implemented (Fig. 2). We first constructed a neighbor-joining phylogenetic tree involving the 93 SBPs. (Fig. 2a). The SBPs were divided into 10 distinct groups according to the phylogenetic analysis, namely, *g1*, *g2*, *g3*, *g4*, *g5*, *g6*, *g7*, *g8*, *g9*, and *g10*. This phylogenetic relationship was further confirmed by the maximum likelihood analysis showing that each group was supported by a bootstrap value > 60% (Fig. 2b). Nine groups (all except *g6*) contained *A. thaliana* SPLs, which is consistent with previous results [14, 40]. In addition, for the groups containing *AtSPL* genes, the Euphorbiaceae *SBP* genes were often close together, while the *A. thaliana SBP* genes were also close together. The protein characteristics of each group are summarized in Table 2. The exon number in each group exhibited a uniform tendency that was consistent with protein length (Fig. 2a).

**Table 2:** The physicochemcial properties of 10 Euphorbiaceae SBP groups

Groups	Mean Length (aa)	Mean Mw	Mean Pi	Target site
g1	304.7	34,075.1	8.95	None
g2	782.7	87,961.2	6.52	None
g3	181.1	20,208.9	8.55	3’UTR
g4	1072	118,801.4	8.82	None
g5	1009.2	111,898.3	6.86	None
g6	403	44,980.9	7.97	CDS
g7	483.3	52,934.7	9.24	CDS
g8	374.3	39,878.7	9.24	CDS
g9	376.2	41,260.6	8.66	CDS
g10	512.5	56,049.3	7.55	CDS

**Fig. 3:**
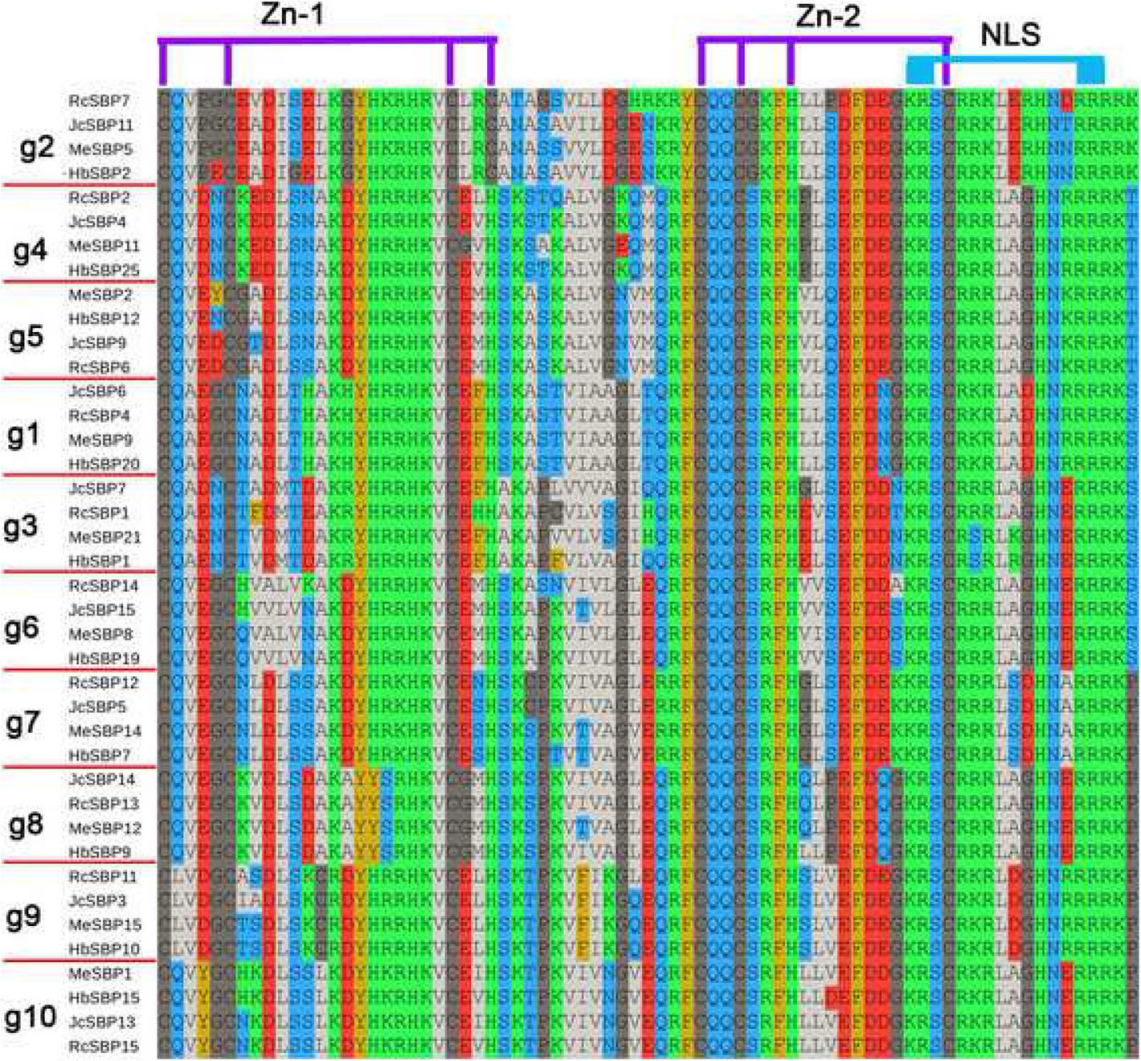
The multiple alignment of SBP-specific domain. One gene in each group for per species was chosen. Zn-1, Zn-2 and one NLS are highlighted on the top

We also conducted multiple sequence alignment for the conserved SBP-specific domain, which contained approximately 75 aa. Due to high structural similarity, we selected only one *SBP* gene per species per group for better visualization. All SBP-specific domains contained two zinc finger motifs and one nuclear localization signal (NLS) motif (Fig. 3). Nevertheless, the first zinc finger motif for *g2* (Cys-Cys-Cys-Cys) was different from that in the other groups (Cys-Cys-Cys-His). For all the members of the 10 groups, compared with the first zinc finger, there was no structural difference in the second zinc finger (which was typically Cys-Cys-His-Cys). Moreover, each group had its own sequence features. For example, the second amino acid residue in *g9* was L, while the fifth amino acid residue was K in *g4* and G in its sister group *g5*.

### Gene structure and conserved motif analysis

**Fig. 4:**
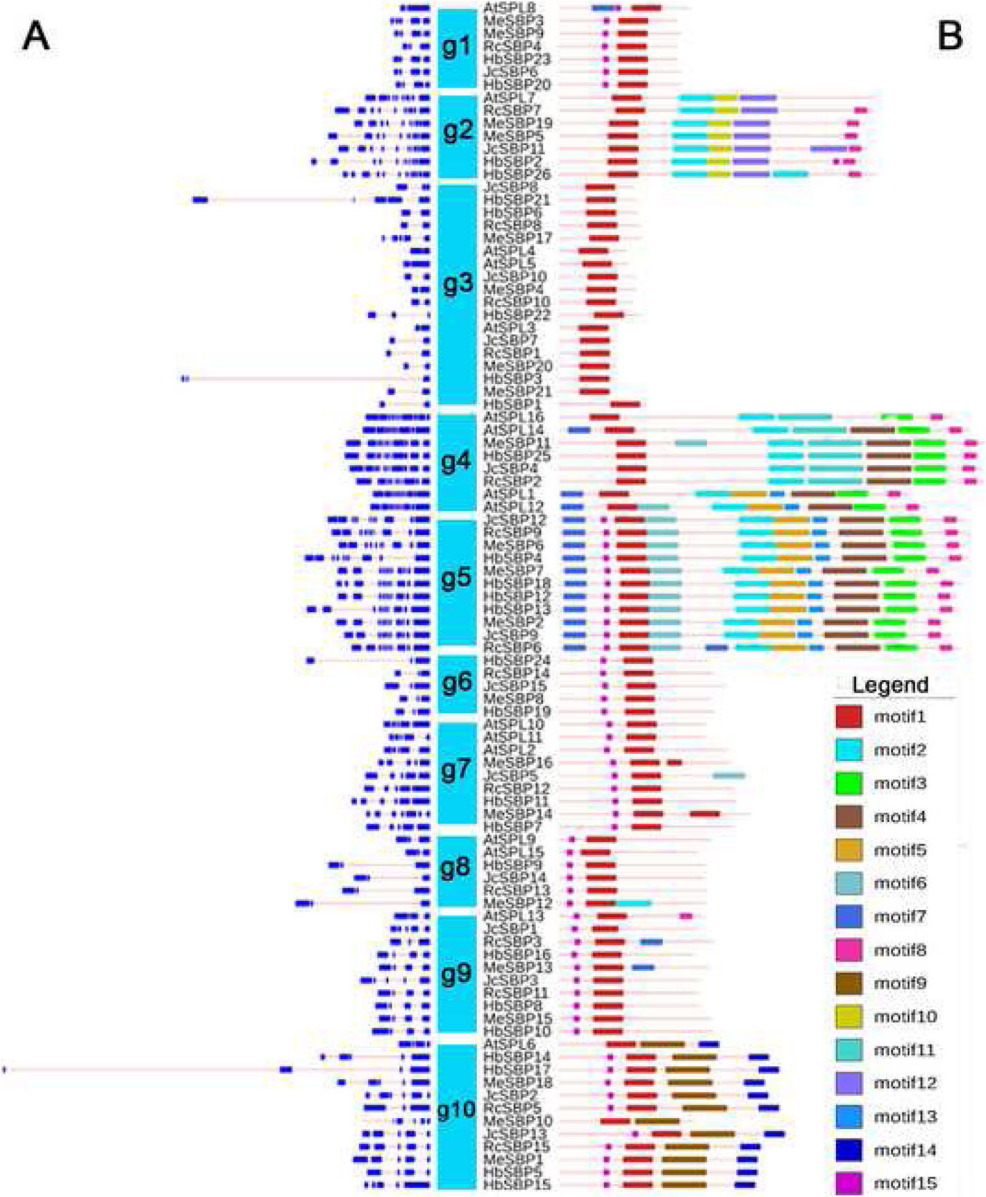
*SBP* gene structures and motifs. Exons are indicated by blue box; introns are indicated by pink lines; UTR sequences are indicated by black boxes. The motifs are highlighted in different colored boxes with numbers 1 to 15. The phylogenetic groups of *g1* to *g10* are indicated in the middle. **a** Schematic representation of intron-exon composition of Euphorbiaceae *SBP* genes. **b** Schematic representation of conserved motifs of Euphorbiaceae SBP transcription factors

We further examined the structures of all *SBP* genes, comprising 77 in Euphorbiaceae and 16 in *A. thaliana* (Fig. 4a). The structural patterns were similar within each group but distinct between any two groups. In addition, the intron lengths of *AtSPL* genes were shorter than those in Euphorbiaceae genes. To identify the structural similarities and differences in SBPs between groups, a conserved motif analysis was performed. A total of 15 conserved motifs, including the SBP-specific domain (motif1), were found (Fig. 4b, Additional file 2: Fig. S1). The motif number was consistent with the protein length (Fig. 4b); the proteins in *g2/4/5* were rich in motifs, sharply contrasting with the proteins in *g3*, which had only one motif. Some motifs were conserved across groups of different length ranges. For example, motif15 was shared for each middle-sized group and long-sized *g5*. Some motifs were group-specific: motif9 and motif14 were unique to *g10*, which was different from other middle-sized groups that contained only 2–3 motifs. Moreover, *g4* and *g5* shared many motifs, while motif5/13/4 were *g5*-specific and motif6 was *g4*-specific. Among the long-sized groups, *g2* exhibited many differences in motifs compared to *g4* and *g5*. In addition, *g5* always contained both Ankyrin (ANK) and transmembrane regions, and the *g5* proteins may be involved in protein–protein interactions.

### Chromosomal locations and gene duplication events

**Fig. 5:**
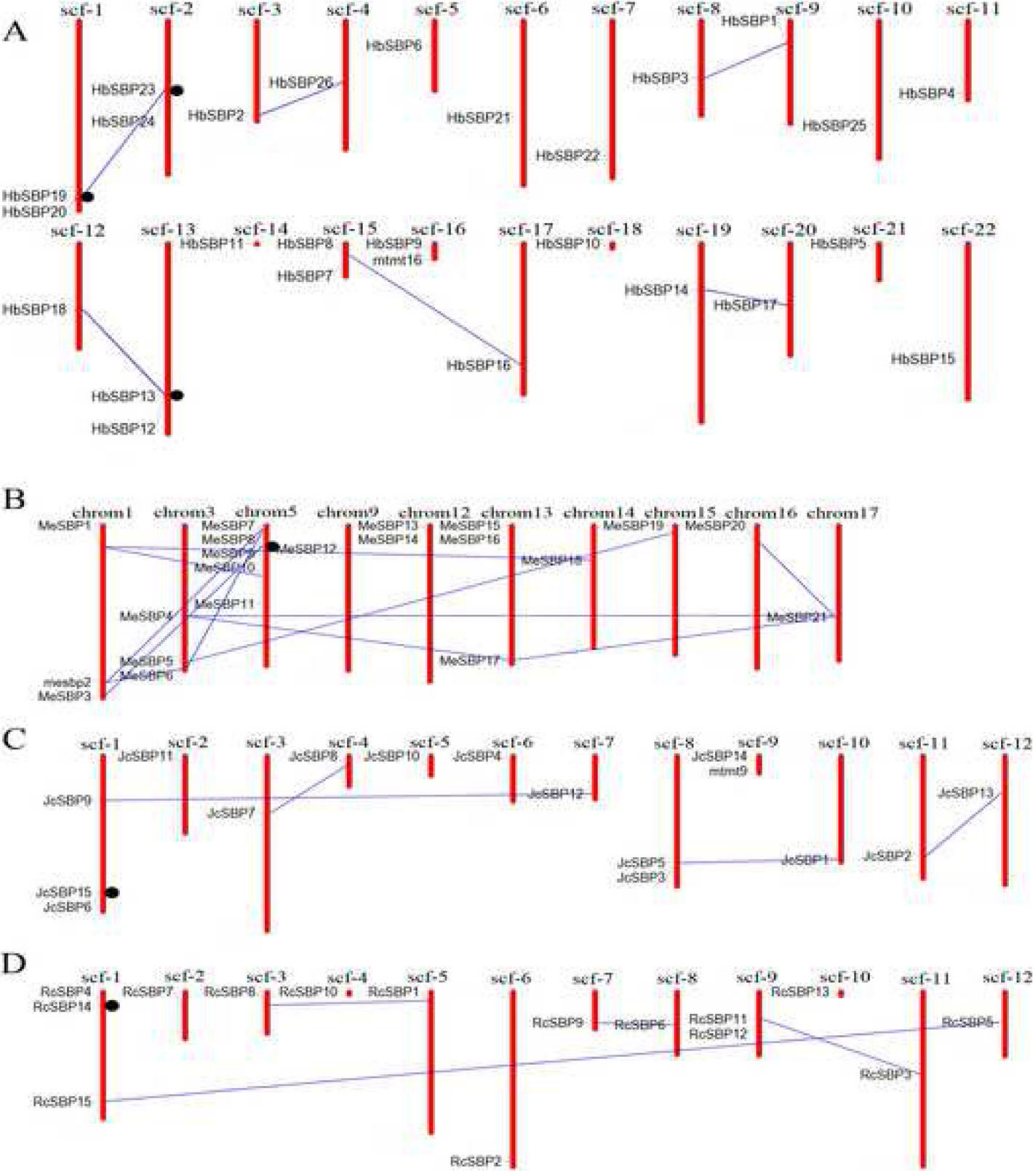
Chromosomal locations and gene duplication events of Euphorbiaceae *SBP* genes. For cassava, the sequence number represents the chromosome number. For physic, rubber tree and castor bean, the scaffold numbers are indicated on the top and their detail scaffold IDs are recorded in Additional file 1: Table S3. *SBP* gene pairs from segmental duplications are linked by blue lines; tandem duplications are marked by black circle. Each species are plotted in a unique part of (**a**) rubber tree, (**b**) cassava, (**c**) physic nut, (**d**) castor bean

The chromosomal distribution of the Euphorbiaceae *SBP* genes throughout the four Euphorbiaceae genomes was plotted using MapInspect software. Because of the lack of chromosome-level assembly data for physic nut, castor bean, and rubber tree, we plotted their *SBP* gene distribution at the scaffold level instead of the chromosome level (Fig. 5, Additional file 1: Table S3). Gene duplication events among the Euphorbiaceae *SBP* genes were also examined (Fig. 5, Additional file 1: Table S4.1). MCScan searching combined with micro-fragment comparison was used to find accurate duplicate gene pairs. Based on these two methods, 26 segment duplications were found: 12 in cassava, 6 in rubber tree, 4 in physic nut, and 4 in castor bean (Additional files 1: Table S4.1). The rubber tree contained the largest number of *SBP* genes but a relatively low number of duplications. Imperfect sequencing data partly led to the incomplete linear relationship between the number of duplicate gene pairs and the genome size. Segment duplications made a greater contribution to the Euphorbiaceae *SBP* gene expansions than tandem duplications (Additional file 1: Table S4.2). Six tandem duplication gene pairs were identified (Fig. 5). Interestingly, each *SBP* gene in *g6* had one tandem duplication gene in *g1* (*HbSBP19*-*HbSBP20*, *HbSBP24*-*HbSBP23*, *JcSBP15*-*JcSBP6*, *RcSBP14*-*RcSBP4*, and *MeSBP8*-*MeSBP9*), which suggests that these tandem duplication SBP genes may result in functional differentiation.

**Fig. 6:**
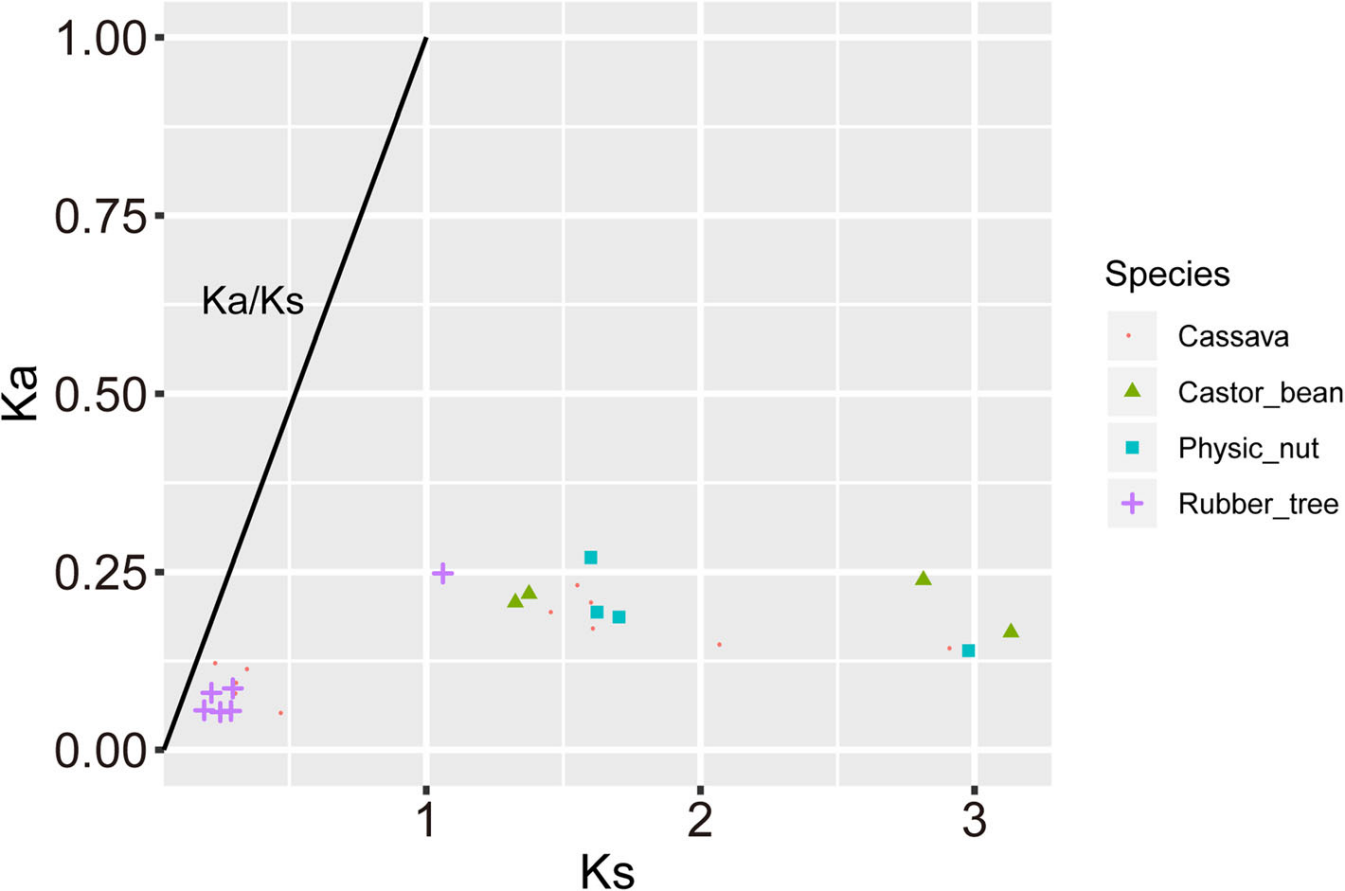
*Ka, Ks* and their ratio. Gene pairs from different species are indicated by different scatter. The x and y axes denote *Ks* and *Ka* for each gene pair and the black line represents *Ka/Ks* ratio = 1. The –S range are the gene pairs whose Ks value less than 1, and the –L range are the gene pairs whose Ks value more than 1. Detailed values of *Ka*, *Ks* and *Ka/Ks* listed in Additional file 1: Table S4

All the predicted segment duplications were found within group, and they support our grouping scheme well. To further understand the evolutionary constraints on the Euphorbiaceae *SBP* genes, synonymous (*Ks*) and nonsynonymous (*Ka*) substitutions per site and their ratio (*Ka/Ks*) were calculated for the segment duplication gene pairs to explore their roles in the expansionary processes of *SBP* genes. The time to a certain duplication event can be calculated using the *Ks* value, as synonymous mutations accumulate at a relatively constant rate over time. Some *Ks* values were < 1 (marked –S) while others were 1–3 (marked –L) (Fig. 6). The bimodal distribution of the *Ks* values indicates that there were two large-scale duplication events. *Ks-S* duplications only existed in cassava and rubber tree, whereas *Ks-L* duplications were shared by all four Euphorbiaceae species (Additional file 1: Table S4.1). Given the *Ks-L* values in rubber tree, the –L duplications are likely to be associated with the triplication event related to all core eudicots [41]. The –L duplications generated branches consisting of conserved Euphorbiaceae genes. All the *Ka-L* values were greater than the *Ka-S* values (Fig. 6). However, the *Ka-L/Ks-L* values were lower than the *Ka-S/Ks-S* ones, which mean that selection pressure on *Ka* was higher than *Ks* for *SBP* genes (Fig. 6). All *Ka/Ks* values were < 0.5 (Fig. 6), suggesting that the Euphorbiaceae SBP-box gene family underwent strong purifying selection to reduce detrimental mutations after duplication.

### Synteny analysis

To explore the evolutionary process of the Euphorbiaceae SBP-box gene family, we conducted a comparative analysis of synteny blocks of genomes among the four Euphorbiaceae species and *A. thaliana* (Additional file 3: Fig. S2). Here, 141 syntenic blocks between Euphorbiaceae species were discovered (Additional file 3: Fig. S2). A high level of synteny relationships were found at both the species level (21/21 *SBP* genes in cassava, 15/15 in physic nut, 13/15 in castor bean, and 17/26 in rubber tree) and group level (all 10 groups were covered). Moreover, no intergroup synteny blocks were found (Additional file 1: Table S5), which is in accordance with the segment duplication results and validated our grouping scheme.

### Prediction of microRNA target sites

We found the target sites of *miR156* either in the CDS or 3’UTR (Table 3). For both *A. thaliana* and Euphorbiaceae, there was a similar ratio (2/1) of with- to without-target *SBP* genes. Long-sized *SBP* genes had no target sites, while both the middle- and short-sized *SBP* genes had target sites located either in CDS or 3’UTR (Table 2). However, one exception was that *g1*, a middle-sized group, contained no *miR156* target (neither in *A. thaliana* nor in the Euphorbiaceae species).

**Table 3:** The *miR156* target information of Euphorbiaceae *SBP* genes

Location	ID	CDS/3’UTR length	Target site	miR site
CDS	JcSBP1	1014	818 GUGCUCUCUCUCUUCUGUCA 837	20 CACGAGAGAGAGAAGACAGU 1
CDS	JcSBP2	1590	1148 GUGCUCUCUCUCUUCUGUCA 1167	20 CACGAGAGAGAGAAGACAGU 1
CDS	JcSBP3	954	683 GUGCUCUCUCUCUUCUGUCA 702	20 CACGAGAGAGAGAAGACAGU 1
CDS	JcSBP5	1443	1154 GUGCUCUCUCUCUUCUGUCA 1173	20 CACGAGAGAGAGAAGACAGU 1
CDS	JcSBP13	1725	1289 GUGCUCUCUCUCUUCUGUCA 130	20 CACGAGAGAGAGAAGACAGU 1
CDS	JcSBP14	1119	230 AAGGGUGUAAAGUGGAUCUGA 250	21 UACCCAUAAUUCAUCUAGACU 1
CDS	JcSBP15	1260	830 GUGCUCUCUCUCUUCUGUCA 849	20 CACGAGAGAGAGAAGACAGU 1
3’UTR	JcSBP7	237	150 CUGCUCUCUCUCUUCUGUCA 169	20 CACGAGAGAGAGAAGACAGU 1
3’UTR	JcSBP8	530	4 UGCUCCCUCUCUUCUGUCAU 23	20 ACGAGAGAGAGAAGACAGUU 1
3’UTR	JcSBP10	214	25 UGCUCCCUCUCUUCUGUCAU 44	20 ACGAGAGAGAGAAGACAGUU 1
CDS	RcSBP3	1149	968 GUGCUCUCUCUCUUCUGUCA 987	20 CACGAGAGAGAGAAGACAGU 1
CDS	RcSBP5	1674	1229 CUGCUCUCUCUCUUCUGUCA 1248	20 CACGAGAGAGAGAAGACAGU 1
CDS	RcSBP11	1155	884 GUGCUCUCUCUCUUCUGUCA 903	20 CACGAGAGAGAGAAGACAGU 1
CDS	RcSBP12	1452	1163 GUGCUCUCUCUCUUCUGUCA 1182	20 CACGAGAGAGAGAAGACAGU 1
CDS	RcSBP13	1134	782 GUGCUCUCUCUCUUCUGUCA 801	20 CACGAGAGAGAGAAGACAGU 1
CDS	RcSBP14	1167	809 GUGCUCUCUCUCUUCUGUCA 828	20 CACGAGAGAGAGAAGACAGU 1
CDS	RcSBP15	1542	1094 GUGCUCUCUCUCUUCUGUCA 1113	20 CACGAGAGAGAGAAGACAGU 1
3’UTR	RcSBP1	214	122 AUGCUCUCUCUCUUCUGUCA 141	20 CACGAGAGAGAGAAGACAGU 1
3’UTR	RcSBP8	235	6 UUGCUCUCUCUCUUCUGUCA 25	20 CACGAGAGAGAGAAGACAGU 1
3’UTR	RcSBP10	325	32 AUGCUCCCUCUCUUCUGUCA 51	20 CACGAGAGAGAGAAGACAGU 1
CDS	HbSBP5	1518	1073 GUGCUCUCUCUCUUCUGUCA 1092	20 CACGAGAGAGAGAAGACAGU 1
CDS	HbSBP7	1446	1160 GUGCUCUCUCUCUUCUGUCA 1179	20 CACGAGAGAGAGAAGACAGU 1
CDS	HbSBP8	1152	881 GUGCUCUCUCUCUUCUGUCA 900	20 CACGAGAGAGAGAAGACAGU 1
CDS	HbSBP9	1125	773 GUGCUCUCUCUCUUCUGUCA 792	20 CACGAGAGAGAGAAGACAGU 1
CDS	HbSBP10	1149	878 GUGCUCUCUCUCUUCUGUCA 897	20 CACGAGAGAGAGAAGACAGU 1
CDS	HbSBP11	1473	1181 GUGCUCUCUCUCUUCUGUCA 1200	20 CACGAGAGAGAGAAGACAGU 1
CDS	HbSBP14	1596	1151 GUGCUCUCUCUCUUCUGUCA 1170	20 CACGAGAGAGAGAAGACAGU 1
CDS	HbSBP15	1500	1073 GUGCUCUCUCUCUUCUGUCA 1092	20 CACGAGAGAGAGAAGACAGU 1
CDS	HbSBP16	1107	917 GUGCUCUCUCUCUUCUGUCA 936	20 CACGAGAGAGAGAAGACAGU 1
CDS	HbSBP17	1674	1229 GUGCUCUCUCUCUUCUGUCA 1248	20 CACGAGAGAGAGAAGACAGU 1
CDS	HbSBP19	1224	818 GUGCUCUCUCUCUUCUGUCA 837	20 CACGAGAGAGAGAAGACAGU 1
CDS	HbSBP24	1197	791 GUGCUCUCUCUCUUCUGUCA 810	20 CACGAGAGAGAGAAGACAGU 1
3’UTR	HbSBP1	263	156 AUGCUCUCUCUCUUCUGUCA 175	20 CACGAGAGAGAGAAGACAGU 1
3’UTR	HbSBP3	266	114 AUGCUCUCUCUCUUCUGUCA 133	20 CACGAGAGAGAGAAGACAGU 1
3’UTR	HbSBP6	389	18 UUGCUCUCUAUCUUCUGUCA 37	20 CACGAGAGAGAGAAGACAGU 1
3’UTR	HbSBP21	2797	18 UUGCUCCCUCUCUUCUGUCA 37	20 CACGAGAGAGAGAAGACAGU 1
3’UTR	HbSBP22	318	19 ACGCUCCCUCUCUUCUGUCA 38	20 CACGAGAGAGAGAAGACAGU 1
CDS	MeSBP1	1518	1073 GUGCUCUCUCUCUUCUGUCA 1092	20 CACGAGAGAGAGAAGACAGU 1
CDS	MeSBP8	1212	818 GUGCUCUCUCUCUUCUGUCA 837	20 CACGAGAGAGAGAAGACAGU 1
CDS	MeSBP10	1050	869 GUGCUCUCUCUCUUCUGUCA 888	20 CACGAGAGAGAGAAGACAGU 1
CDS	MeSBP12	1125	773 GUGCUCUCUCUCUUCUGUCA 792	20 CACGAGAGAGAGAAGACAGU 1
CDS	MeSBP13	1146	875 GUGCUCUCUCUCUUCUGUCA 894	20 CACGAGAGAGAGAAGACAGU 1
CDS	MeSBP14	1467	1178 GUGCUCUCUCUCUUCUGUCA 1197	20 CACGAGAGAGAGAAGACAGU 1
CDS	MeSBP15	1158	881 GUGCUCUCUCUCUUCUGUCA 900	20 CACGAGAGAGAGAAGACAGU 1
CDS	MeSBP16	1437	1151 GUGCUCUCUCUCUUCUGUCA 1170	20 CACGAGAGAGAGAAGACAGU 1
CDS	MeSBP18	1563	1118 GUGCUCUCUCUCUUCUGUCAU 1138	21 CACGAGAGAGAGAAGACAGUU 1
3’UTR	MeSBP4	211	16 AUGCUCCCUCUCUUCUGUCA 35	20 CACGAGAGAGAGAAGACAGU 1
3’UTR	MeSBP17	996	18 UUGCUCCCUCUCUUCUGUCA 37	20 CACGAGAGAGAGAAGACAGU 1
3’UTR	MeSBP20	218	171 GUGCUCUCUCUCGUAUGUCA 190	20 CACGAGAGAGAGAAGACAGU 1
3’UTR	MeSBP21	384	122 AUGCUCUCUAUCUUCUGUCA 141	20 CACGAGAGAGAGAAGACAGU 1

### Tissue expression profiles of *JcSBP* genes

**Fig. 7:**
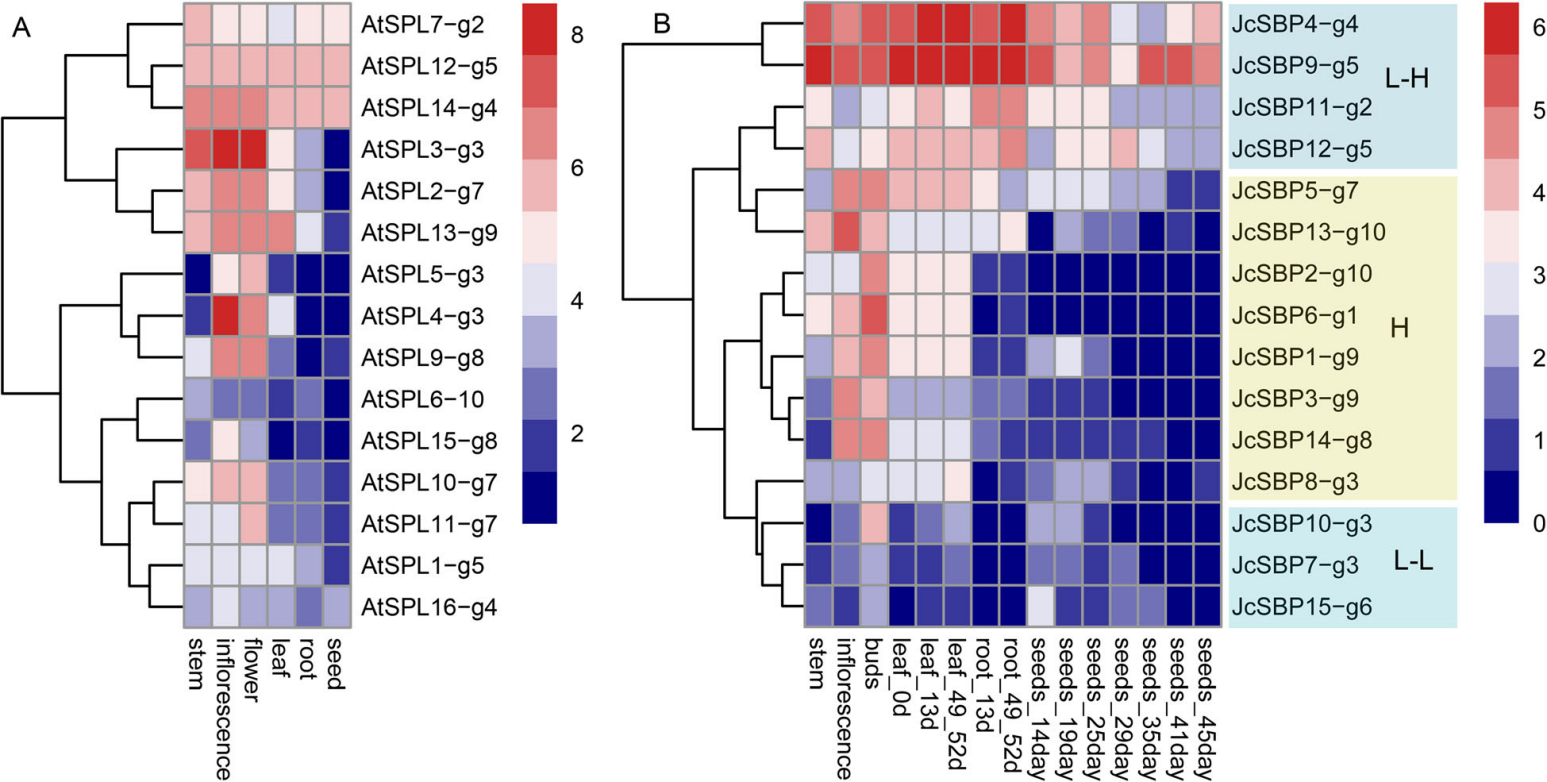
The tissue expression profiles. The tissue expression profiles of *A. thaliana* (**a**). Expression profiles of physic nut *SBP* genes among different tissues and development stages (**b**). The low expression differential groups were highlighted in blue (marked with L), and the high expression differential groups were highlighted in orange (marked with H). The blue groups can be further divided into high expressional and low expressional groups that marked with L-H and L-L respectively

To further illustrate the potential functions of each *SBP* gene, we conducted a comparative analysis of the expression data (from stem, inflorescence, buds, leaf, root, and seed) of physic nut and *A. thaliana* (Fig. 7). Because of the high similarity of *SBP* genes among the four Euphorbiaceae species, the analysis of the *SBP* genes of physic nut is very representative. Hierarchical clustering was used to visualize the global expression profile of the *JcSBP* genes (Fig. 7b). The expression patterns of the *JcSBP* genes could be divided into low differential expression between tissues (*JcSBP4*, *JcSBP9*, *JcSBP11*, *JcSBP12*, *JcSBP10*, *JcSBP7*, and *JcSBP15*) and high differential expression between tissues (*JcSBP5*, *JcSBP13*, *JcSBP2*, *JcSBP6*, *JcSBP1*, *JcSBP3*, *JcSBP14*, and *JcSBP8*). The former could be further divided into low expression genes (*JcSBP10*, *JcSBP7*, and *JcSBP15*) and high expression genes (*JcSBP4*, *JcSBP9*, *JcSBP11*, and *JcSBP12*).

There were significant differences in the expression profiles of *JcSBP* genes between the with- and without-target genes (Fig. 7b). The *JcSBP* genes of *g2/4/5* (long-sized groups) contained no target sites, and they were highly expressed without differential expression between tissues. In contrast, the with-target *JcSBP* genes in the middle-sized groups were highly differentially expressed in different tissues (with high expression in the buds and inflorescences, though several genes also played roles in the stem, leaf, or root). However, the tissue expression differences of the other with-target *JcSBP* genes (in the short-sized groups) were not as significant as the with-target *JcSBP* genes in the middle-sized groups.

The expression patterns of *AtSPL* genes in *g3* and *g10* were significantly different from those in physic nut (Fig. 7). Regarding *g3*, the relative expression intensity of *AtSPL* genes was higher than those in physic nut, and they were highly expressed in more tissues. In contrast, regarding *g10*, the relative expression intensity of *JcSBP* genes was higher than *AtSPL* genes. The expression signal of *AtSPL6* was barely observable. However, *JcSBP2* and *13* were redundantly expressed in the stem, inflorescence, and root.

### Stress response expression profiles of *JcSBP* genes

**Fig. 8:**
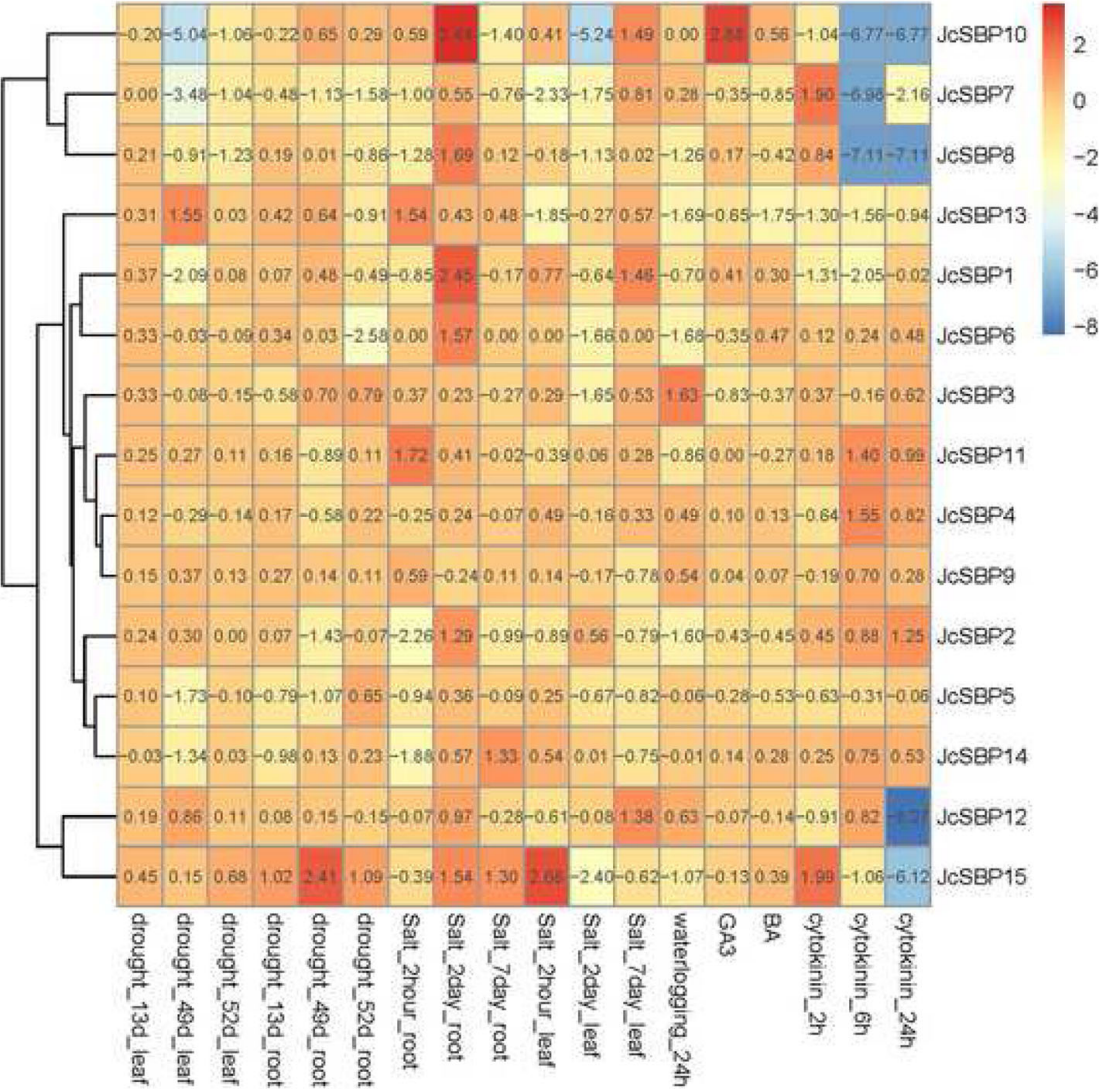
Expression profiles of physic nut *SBP* genes in response to abiotic stress and hormone stress treatments. The numerical values in different color scales represent log2 transform of the ratio of the experimental group and control group in a specific treatment condition

To further explore the possible physiological processes in which Euphorbiaceae *SBP* genes participate, the expression levels in physic nut in response to various abiotic stresses (salt, drought, and waterlogging) and hormonal treatments (gibberellin 3 [GA3], 6-benzylaminopurine [BA], and cytokinin) were obtained. Log2 transformations of the ratio of the treatment group data to their corresponding control group data are displayed in Fig. 8; log2 transformed values > 1 or < − 1 were viewed as representing differential expression.

First, in response to drought (Fig. 8), *JcSBP7* and *JcSBP10* showed > 4-fold decreased expression in the leaves. In the roots, *JcSBP7*, *JcSBP6*, *JcSBP2*, and *JcSBP5* were down-regulated, while *JcSBP15* was up-regulated under all drought treatments. Second, in response to salt (Fig. 8), eight *JcSBP* genes (*JcSBP1*, *JcSBP2*, *JcSBP6*, *JcSBP8*, *JcSBP10*, *JcSBP11*, *JcSBP13*, and *JcSBP15*) were up-regulated in the roots. Six *JcSBP* genes (*JcSBP2*, *JcSBP6*, *JcSBP8*, *JcSBP7*, *JcSBP10*, and *JcSBP14*) showed > 2-fold decreased expression in the roots. In the leaves, there were six down-regulated *JcSBP* genes (*JcSBP10*, *JcSBP7*, *JcSBP13*, *JcSBP6*, *JcSBP3*, and *JcSBP15*) and four up-regulated *JcSBP* genes (*JcSBP10*, *JcSBP1*, *JcSBP12*, and *JcSBP15*), while *JcSBP10* and *JcSBP15* showed both up- and down-regulated patterns. Third, in response to waterlogging treatment, several *JcSBP* genes were down-regulated (*JcSBP8*, *JcSBP13*, *JcSBP6*, *JcSBP2*, and *JcSBP15*) or up-regulated (*JcSBP3*).

We further assessed the expression level of *JcSBP* genes in response to GA3, BA, and cytokinin treatments (Fig. 8). Compared to the control groups, *JcSBP10* was increased almost 8-fold in response to GA3. *JcSBP13* decreased by > 2-fold in response to BA. Compared with the response to GA3 and BA, more *JcSBP* genes were up-regulated in response to cytokinin. Five *JcSBP* genes (*JcSBP10*, *JcSBP8*, *JcSBP13*, *JcSBP1*, and *JcSBP12*) decreased in response to cytokinin and three increased (*JcSBP11*, *JcSBP4*, and *JcSBP2*). Additionally, two *JcSBP* genes (*JcSBP7* and *JcSBP15*) displayed both up- and down-regulated expression.

## Discussion

In view of their excellent agricultural traits, several Euphorbiaceae species have become important food sources or industrial raw materials. Cassava [27, 28], physic nut [29, 30], castor bean [25, 42], and rubber tree [31] have been widely domesticated and cultivated. The continuously increasing quantity of genome sequencing data, genetic linkage maps, and abundance of high-throughput transcriptome sequencing data make further exploration of gene functions in non-model plants like Euphorbiaceae species possible. Previous studies on *SBP* genes have revealed their crucial roles in plant development, especially in flower development, signal transduction, and defense processes [5–10]. However, the functions of Euphorbiaceae SBPs are still unknown. In this study, genome-wide analyses (including the analyses of the evolutionary trajectory, *miR156* regulation, and expression profiles) of the Euphorbiaceae SBP-box gene family were conducted to shed new light on Euphorbiaceae *SBP* genes.

The phylogenetic relationships, synteny analysis, and tissue expression profiles showed that the *SBP* genes of Euphorbiaceae and *A. thaliana* are similar in structure, evolutionary trajectory, and functions. In light of the high similarity between *SBP* genes of Euphorbiaceae and *A. thaliana*, we can predict the functions of some of the *SBP* genes of Euphorbiaceae based on the well-studied *AtSPL* genes. Regarding the long-sized groups, *AtSPL7* (in *g2*) has been reported to be related to Cu homeostasis in *A. thaliana*, and it regulates the expression of Cu-responsive genes and is considered to be a central regulator of copper homeostasis [43, 44]. The gene that is homologous to *AtSPL7* was conserved in Euphorbiaceae and, similar to *A. thaliana*, it exhibited significantly high expression in the roots. Mutations of *AtSPL14* (in *g4*) result in resistance to the fungal toxin fumonisin B1 [45]. *AtSPL1* and *AtSPL12* (in *g5*) play redundant roles in thermotolerance at the reproductive stage [9].

Regarding the middle-sized groups (g1/6/7/8/9/10), one of their remarkable characteristics is that they can be regulated by miR156 (all except *g1*). Due to regulation by miR156, these SBP genes play critical roles in plant development. *AtSPL13* (in *g9*) has been implicated in delaying leaf outgrowth during germination [46]. *AtSPL2, AtSPL10*, and *AtSPL11* (in *g7*) affect the morphological features associated with phase change [7]. *AtSPL9* and *AtSPL15* (in *g8*) play redundant roles in reproductive transition and vegetative phase change [8, 47]. *AtSPL8* (in *g1*) is related to seed formation, root development, and petal trichome [48, 49]. As in *A. thaliana*, all the middle-sized *JcSBP* genes were differentially expressed between different tissues and exhibited high intensity expression, which suggests that they may be involved in different physiological processes and play critical roles in plant development and reproduction.

As we know, *A. thaliana* is monoecious, while physic nut is diecious; *A. thaliana* is a kind of biennial herb, while physic nut is a kind of perennial woody plant. It is worth exploring the functions of Euphorbiaceae *SBP* genes regarding the flowering process, phase transformation, seed development, etc. We found that the expression patterns of the *SBP* genes in *g3* were significantly different between *A. thaliana* and physic nut, and there may be functional differences between them. In addition, regarding *g10*, the tissue expression profiles of *A. thaliana* were significantly different from those of physic nut in both relative expression intensity and the differential expression between different tissues. Moreover, *g6* was absent from *A. thaliana* but conserved in Euphorbiaceae, and it was highly expressed in seeds and exhibited a relatively high response to salt, drought, and cytokinin. These results suggest that there may be some new functions or regulatory forms of *SBP* genes in Euphorbiaceae, and understanding these genes is helpful to further reveal the physiological regulation processes in Euphorbiaceae.

Sometimes plants are cultivated for their roots, sometimes for their seeds, and sometimes for their fruits. The formation of different tissues and organs may be related to different regulatory processes. Our study suggests that some SBP genes are differentially expressed in different tissues and organs, and may be associated with specific physiological processes. For example, physic nut and castor bean are cultivated for their seeds, so flower development and seed formation are important for a higher crop production. Both middle- and small-sized SBP genes are related to inflorescence or bud development according to their tissue expression profiles (Fig. 7b). In addition, several SBP genes were found to be related to seed development, such as JcSBP5/13/1/8, which express relative high in seeds (Fig. 7b). On the other hand, unlike physic nut and castor bean, cassava is cultivated for its roots, and JcSBP5/13 are highly expressed in the roots (Fig. 7b). Therefore, increasing the study of these SBP genes may contribute to the deeper understanding of specific physiological processes and subsequent agricultural genetics studies.

## Conclusions

SBP-box genes encode a series of plant-specific TFs, which have been identified and characterized in several species. Significant progress has been achieved regarding the identification of the functions of some *SBP* genes in several species, but little attention has been paid to non-model plants. In the present study, we identified 77 putative *SBP* genes in the genomes of four Euphorbiaceae species. From the results of the phylogeny analysis, we divided the Euphorbiaceae *SBP* genes into 10 independent groups, and the subsequent results regarding the structural analysis and the distribution of duplication gene pairs supported our grouping scheme. The genome comparison indicated that segment duplication played crucial roles in Euphorbiaceae *SBP* gene expansion, and all the duplication gene pairs were subjected to purify selection. In addition, two-thirds of Euphorbiaceae *SBP* genes may be regulated by *miR156*, and these miR-regulated genes all belonged to the middle- or short-sized groups. Comparative synteny analysis between the genomes of five species (including *A. thaliana*) showed that a large number of *SBP* genes were located in syntenic regions, implying that these *SBP* genes probably come from common ancestors. Furthermore, to illustrate the probable functions of these *SBP* genes, we conducted a comparative analysis of the expression profiles of *JcSBP* and *AtSPL* genes in various tissues/organs. Most miR-regulated *JcSBP* genes were more differentially expressed than miR-nonregulated *JcSBP* genes. *G6* is conserved in Euphorbiaceae but not in *A. thaliana*, and we assume that it is functionally active as it was highly expressed in the buds and stems. However, the short-sized *JcSBP* genes were not as active as their homologous *AtSPL* genes, indicating there may be some functional differences between *A. thaliana* and Euphorbiaceae. Lastly, many *JcSBP* genes were up- or down-regulated in response to certain abiotic or phytohormone stresses, implying that they may be involved in the responses to various stresses or in physic nut development. Our data provide valuable information for further functional studies of Euphorbiaceae *SBP* genes. The flowering mechanism between *A. thaliana* and Euphorbiaceae and the high demand for increases in crop yield make the exploration of Euphorbiaceae *SBP* genes highly valuable.

## Methods

### Data sources

Genomic and proteomic sequences were obtained from the Phytozome portal for cassava (manihot_esculenta_v6, JGI; https://phytozome.jgi.doe.gov/pz/portal.html), National Center for Biotechnology Information (NCBI) for castor bean (JCVI_RCG_v1.1; https://www.ncbi.nlm.nih.gov/), NCBI for rubber tree (ASM165405v1; https://www.ncbi.nlm.nih.gov/), and NCBI for physic nut (JatCur_1.0; https://www.ncbi.nlm.nih.gov/). The *A. thaliana* genomic and proteomic sequences were obtained from TAIR (TAIR10 release; https://www.arabidopsis.org/). Gene expression data for physic nut were obtained from the NCBI (https://www.ncbi.nlm.nih.gov/).

### Identification, characterization, and phylogenetic analysis

Both HMM [50] and BLASTP [51] searches were performed to accurately identify the SBP TFs in the Euphorbiaceae species. The well-characterized *A. thaliana* SBP protein sequences were used as queries for BLASTP searches (e-value ≤1e-10). The SBP-specific HMM profile (PF03110) was used for queries, and the HMMER toolkit was used in the HMM searches. The conserved SBP-specific domain was confirmed using Simple Modular Architecture Research Tool (SMART) [52] (http://smart.embl-heidelberg.de/), and the incomplete SBP-specific domains were discarded. In the cases involving multiple transcripts of the same gene, a dot followed by a serial number was added at the end of each name. The physicochemical properties, including protein length, molecular weight (MW), and isoelectric point (Pi), for the identified SBP proteins were predicted using the ExPASy Proteomics Server (https://prosite.expasy.org/) [53]. Multiple sequence alignment of SBP protein sequences was performed by Multiple Sequence Comparison by Log-Expectation (MUSCLE) in MEGA v7.0 [54]. A neighbor-joining tree was constructed using MEGA v7.0. The maximum likelihood tree was generated using the PAUP* program, employing the JTT substitution model and 100 bootstrap replicates [55].

### Conserved motifs and gene structure analysis

The online Multiple Expectation Maximization for Motif Elucidation (MEME) toolkit was used to identify additional motifs (http://meme-suite.org/) [56], which were conserved and located outside the SBP-specific domain region. All SBP protein sequences were used for the queries. The parameters were set as follows: minimum width was 6, maximum width was 150, motif number was 15, and minimum number of sites was 2. Both *SBP* gene sequences and the corresponding coding sequences were uploaded to the online Gene Structure Display Server (GSDS v2.0; http://gsds.cbi.pku.edu.cn/) to obtain intron/exon structure information [57].

### Chromosomal localization

A gene location map for each Euphorbiaceae species based on the chromosomal position of each *SBP* gene was generated by MapInspect (https://mapinspect.software.informer.com/). *SBP* gene locations of cassava were mapped into chromosomes, and *SBP* gene locations of the other three species were mapped into scaffolds due to their incomplete genome assembly information.

### Detection of gene duplication events and synteny relationships

Duplicated gene pairs derived from tandem or segmental duplication were identified according to the method described in the Plant Genome Duplication Database [58]. An all-against-all BLASTP comparison (e-value ≤1e-10) provided gene pairs for syntenic clustering using MCScan v1.1 (e-value ≤1e-10) [59]. Segment duplication was also predicted by the micro-fragment comparison method. The *SBP* duplicate gene pairs from the above analysis were again examined by BLASTP (e-value ≤1e-10), and all the *SBP* genes obtained from the above analysis were used as anchors of micro-fragments generated by the collection of 20 upstream and 20 downstream coding genes. Tandem duplications were identified if two *SBP* genes were next to each other or they had one unrelated gene between them [60].

### Estimation of synonymous (*Ks*) and nonsynonymous (*Ka*) substitutions per site and their ratio (*Ka/Ks*)

*SBP* gene pairs caused by segmental duplication were used to estimate *Ka*, *Ks*, and their ratio. Coding sequences from segmentally duplicated *SBP* gene pairs were aligned using webPRANK (https://www.ebi.ac.uk/goldman-srv/webprank/) [61]. KaKs_Calculator v2.0 [62] was used to compute *Ka*, *Ks*, and *Ka/Ks*. All the counting processes followed the YN model [63] (a simple model of voting). The *Ka/Ks* value can reveal the selective pressure of duplicated genes [64], and the *Ks* value can reflect the divergence time for duplication events. All-against-all BLASTP searches (e-value ≤1e-10) were conducted to investigate the synteny relationships of the proteomes of the four Euphorbiaceae species and *A. thaliana*. The synteny blocks were then calculated using MCScan v1.1 [59], and the synteny relationships were visualized using Circos v0.69–5 [65].

### MicroRNA target prediction

*MiR156* and *miR157* were combined into the *miR156* family in miRBase (https://www.mirbase.org/) [66], due to their highly similar structures. The well-characterized *miR156* mature sequences from miRBase were set as the background data to search against the mRNA sequences of Euphorbiaceae *SBP* genes using psRNATarget program (http://plantgrn.noble.org/v1_psRNATarget/) [67] with default parameters. The detailed positions of miRNA (located in the CDS or 3’UTR region) were further determined on the basis of the locations of target sites and the CDS length.

### Expression analysis

*SBP* gene expression data in six tissues (stem, inflorescence, bud, root, and seed) and under various treatments (gibberellin [GA3], 6-benzylaminopurine [BA], cytokinin, high salt concentration, drought, and waterlogging) of the four Euphorbiaceae species were retrieved from NCBI (https://www.ncbi.nlm.nih.gov/). *A. thaliana* expression data were obtained from TAIR (TAIR10 release; https://www.arabidopsis.org/). All data were analyzed using the Tuxedo suite (TopHat and Cufflinks; http://post.queensu.ca/~rc91/NGS/TuxedoTutorial.html) [68], and they were then upper quartile normalized and log2 transformed. The gene expression profiles were displayed in heatmaps using the R package pheatmap [69].

## Supplementary information

**Additional file 1. **This file contains the additional tables (Table S1-S5) associated with the manuscript. Table numbers and titles were listed as follows: **Table S1:** The information of Euphorbiaceae *SBP* genes. **Table S2:** The protein physicochemical properties of Euphorbiaceae SBP proteins. **Table S3:** The parallel table of scaffold IDs and serial number. **Table S4:** The information of duplications. **Table S5:** The identified synteny relationships between Euphorbiaceae species.

**Additional file 2: Fig. S1:** The sequence logos of 15 motifs.

**Additional file 3: Fig. S2:** The synteny relationships among Euphorbiaceae and *A. thaliana*.

## Data Availability

The datasets supporting the conclusions of this article are included in the article and in its additional files.

## Abbreviations

ANK: Ankyrin
BA: 6-Benzylaminopurine
CDS: Coding DNA sequence
GA: Gibberellins
HMM: Hidden Markov Model
Ka: Nonsynonymous substitutions per non-synonymous site
Ks: Synonymous substitutions per synonymous site
MEME: Multiple Expectation Maximization for Motif Elucidation
ML: Maximum likelihood
MW: Molecular weight
NJ: Neighbor-joining
NLS: Nuclear localization signal
Pi: Isoelectric point
UTR: Untranslated regions

## References

[1] Gong W, Shen YP, Ma LG, Pan Y, Du YL, Wang DH (2004). Genome-wide ORFeome cloning and analysis of *Arabidopsis* transcription factor genes. Plant Physiol.

[2] Klein J, Saedler H, Huijser P (1996). A new family of DNA binding proteins includes putative transcriptional regulators of the *Antirrhinum majus* floral meristem identity gene *SQUAMOSA*. Mol Gen Genet.

[3] Guo AY, Zhu QH, Gu X, Ge S, Yang J, Luo J (2008). Genome-wide identification and evolutionary analysis of the plant specific SBP-box transcription factor family. Gene..

[4] Riese M, Hohmann S, Saedler H, Munster T, Huijser P (2007). Comparative analysis of the SBP-box gene families in *P. patens* and seed plants. Gene..

[5] Yamaguchi A, Wu MF, Yang L, Wu G, Poethig RS, Wagner D (2009). The microRNA-regulated SBP-box transcription factor SPL3 is a direct upstream activator of LEAFY, FRUITFULL, and APETALA1. Dev Cell.

[6] Jung JH, Lee HJ, Ryu JY, Park CM (2016). SPL3/4/5 integrate developmental aging and photoperiodic signals into the FT-FD module in *Arabidopsis* flowering. Mol Plant.

[7] Shikata M, Koyama T, Mitsuda N, Ohme-Takagi M (2009). *Arabidopsis* SBP-box genes *SPL10*, *SPL11* and *SPL2* control morphological change in association with shoot maturation in the reproductive phase. Plant Cell Physiol.

[8] Schwarz S, Grande AV, Bujdoso N, Saedler H, Huijser P (2008). The microRNA regulated SBP-box genes *SPL9* and *SPL15* control shoot maturation in *Arabidopsis*. Plant Mol Biol.

[9] Chao LM, Liu YQ, Chen DY, Xue XY, Mao YB, Chen XY (2017). *Arabidopsis* transcription factors SPL1 and SPL12 confer plant Thermotolerance at reproductive stage. Mol Plant.

[10] Zhang H, Zhao X, Li J, Cai H, Deng XW, Li L (2014). MicroRNA408 is critical for the *HY5-SPL7* gene network that mediates the coordinated response to light and copper. Plant Cell.

[11] Manning K, Tor M, Poole M, Hong Y, Thompson AJ, King GJ (2006). A naturally occurring epigenetic mutation in a gene encoding an SBP-box transcription factor inhibits tomato fruit ripening. Nat Genet.

[12] Wang S, Wu K, Yuan Q, Liu X, Liu Z, Lin X (2012). Control of grain size, shape and quality by OsSPL16 in rice. Nat Genet.

[13] Luo L, Li W, Miura K, Ashikari M, Kyozuka J (2012). Control of tiller growth of rice by OsSPL14 and Strigolactones, which work in two independent pathways. Plant Cell Physiol.

[14] Cardon G, Höhmann S, Klein J, Nettesheim K, Saedler H, Huijser P (1999). Molecular characterisation of the *Arabidopsis* SBP-box genes. Gene..

[15] Cardon GH, Höhmann S, Nettesheim K, Saedler H, Huijser P (1997). Functional analysis of the *Arabidopsis thaliana* SBP-box gene *SPL3*: a novel gene involved in the floral transition. Plant J.

[16] Birkenbihl RP, Jach G, Saedler H, Huijser P (2005). Functional dissection of the plant-specific SBP-domain: overlap of the DNA-binding and nuclear localization domains. J Mol Biol.

[17] Voinnet O (2009). Origin, biogenesis, and activity of plant microRNAs. Cell.

[18] Rogers K, Chen X (2013). Biogenesis, turnover, and mode of action of plant microRNAs. Plant Cell.

[19] Addo-Quaye C, Eshoo TW, Bartel DP, Axtell MJ (2008). Endogenous siRNA and miRNA targets identified by sequencing of the *Arabidopsis* degradome. Curr Biol.

[20] Rhoades MW, Reinhart BJ, Lim LP, Burge CB, Bartel B, Bartel DP (2002). Prediction of plant MicroRNA targets. Cell..

[21] Axtell MJ, Bowman JL (2008). Evolution of plant microRNAs and their targets. Trends Plant Sci.

[22] Arshad M, Feyissa BA, Amyot L, Aung B, Hannoufa A (2017). MicroRNA156 improves drought stress tolerance in alfalfa (*Medicago sativa*) by silencing *SPL13*. Plant Sci.

[23] Wu G, Park MY, Conway SR, Wang JW, Weigel D, Poethig RS (2009). The sequential action of miR156 and miR172 regulates developmental timing in *Arabidopsis*. Cell..

[24] Wu G, Poethig RS (2006). Temporal regulation of shoot development in *Arabidopsis thaliana* by miR156 and its target *SPL3*. Development..

[25] Ogunniyi DS (2006). Castor oil: a vital industrial raw material. Bioresour Technol.

[26] Mubofu EB (2016). Castor oil as a potential renewable resource for the production of functional materials. Sustain Chem Process.

[27] Balat M, Balat H (2009). Recent trends in global production and utilization of bio-ethanol fuel. Appl Energy.

[28] Schmitz PM, Kavallari A (2009). Crop plants versus energy plants--on the international food crisis. Bioorg Med Chem.

[29] King AJ, He W, Cuevas JA, Freudenberger M, Ramiaramanana D, Graham IA (2009). Potential of *Jatropha curcas* as a source of renewable oil and animal feed. J Exp Bot.

[30] Achten WMJ, Mathijs E, Verchot L, Singh VP, Aerts R, Muys B (2007). *Jatropha* biodiesel fueling sustainability?. Biofuels Bioprod Biorefin.

[31] Lau NS, Makita Y, Kawashima M, Taylor TD, Kondo S, Othman AS (2016). The rubber tree genome shows expansion of gene family associated with rubber biosynthesis. Sci Rep.

[32] Li J, Hou H, Li X, Xiang J, Yin X, Gao H (2013). Genome-wide identification and analysis of the SBP-box family genes in apple (*Malus x domestica* Borkh.). Plant Physiol Biochem.

[33] Mao H-D, Yu L-J, Li Z-J, Yan Y, Han R, Liu H (2016). Genome-wide analysis of the SPL family transcription factors and their responses to abiotic stresses in maize. Plant Gene.

[34] Chan AP, Crabtree J, Zhao Q, Lorenzi H, Orvis J, Puiu D (2010). Draft genome sequence of the oilseed species *Ricinus communis*. Nat Biotechnol.

[35] Tang C, Yang M, Fang Y, Luo Y, Gao S, Xiao X (2016). The rubber tree genome reveals new insights into rubber production and species adaptation. Nat Plants.

[36] Wu P, Zhou C, Cheng S, Wu Z, Lu W, Han J (2015). Integrated genome sequence and linkage map of physic nut (*Jatropha curcas* L.), a biodiesel plant. Plant J.

[37] Prochnik S, Marri PR, Desany B, Rabinowicz PD, Kodira C, Mohiuddin M (2012). The cassava genome: current progress, future directions. Trop Plant Biol.

[38] Zhang SD, Ling LZ (2014). Genome-wide identification and evolutionary analysis of the SBP-box gene family in castor bean. PLoS One.

[39] Ren R, Wang H, Guo C, Zhang N, Zeng L, Chen Y (2018). Widespread whole genome duplications contribute to genome complexity and species diversity in angiosperms. Mol Plant.

[40] Yang Z, Wang X, Gu S, Hu Z, Xu H, Xu C (2008). Comparative study of SBP-box gene family in *Arabidopsis* and rice. Gene..

[41] Jiao Y, Leebens-Mack J, Ayyampalayam S, Bowers JE, McKain MR, McNeal J (2012). A genome triplication associated with early diversification of the core eudicots. Genome Biol.

[42] Fidias P, Grossbard M, Lynch TJ (2002). A phase II study of the Immunotoxin N901-blocked ricin in small-cell lung cancer. Clin Lung Cancer.

[43] Lännenpää M, Jänönen I, Hölttä-Vuori M, Gardemeister M, Porali I, Sopanen T (2004). A new SBP-box gene *BpSPL1* in silver birch (*Betula pendula*). Physiol Plant.

[44] Yamasaki H, Hayashi M, Fukazawa M, Kobayashi Y, Shikanai T (2009). *SQUAMOSA* promoter binding protein–Like7 is a central regulator for copper homeostasis in *Arabidopsis*. Plant Cell.

[45] Stone JM, Liang X, Nekl ER, Stiers JJ (2005). *Arabidopsis* AtSPL14, a plant-specific SBP-domain transcription factor, participates in plant development and sensitivity to fumonisin B1. Plant J.

[46] Martin RC, Asahina M, Liu P-P, Kristof JR, Coppersmith JL, Pluskota WE (2010). The microRNA156 and microRNA172 gene regulation cascades at post-germinative stages in *Arabidopsis*. Seed Sci Res.

[47] Hyun Y, Richter R, Vincent C, Martinez-Gallegos R, Porri A, Coupland G (2016). Multi-layered regulation of SPL15 and cooperation with SOC1 integrate endogenous flowering pathways at the *Arabidopsis* shoot meristem. Dev Cell.

[48] Unte US, Sorensen A-M, Pesaresi P, Gandikota M, Leister D, Saedler H (2003). *SPL8*, an SBP-box gene that affects pollen sac development in *Arabidopsis*. Plant Cell.

[49] Zhang Y, Schwarz S, Saedler H, Huijser P (2007). *SPL8*, a local regulator in a subset of gibberellin-mediated developmental processes in *Arabidopsis*. Plant Mol Biol.

[50] Stanke M, Waack S (2003). Gene prediction with a hidden Markov model and a new intron submodel. Bioinformatics.

[51] Mount DW (2007). Using the Basic Local Alignment Search Tool (BLAST). Cold Spring Harb Protoc.

[52] Schultz J, Milpetz F, Bork P, Ponting CP (1998). SMART, a simple modular architecture research tool: identification of signaling domains. PNAS..

[53] Artimo P, Jonnalagedda M, Arnold K, Baratin D, Csardi G, de Castro E (2012). ExPASy: SIB bioinformatics resource portal. Nucleic Acids Res.

[54] Kumar S, Stecher G, Tamura K (2016). MEGA7: molecular evolutionary genetics analysis version 7.0 for bigger datasets. Mol Biol Evol.

[55] Swofford DL. PAUP*. Phylogenetic analysis using parsimony (*and other methods). 2003.

[56] Bailey TL, Boden M, Buske FA, Frith M, Grant CE, Clementi L (2009). MEME SUITE: tools for motif discovery and searching. Nucleic Acids Res.

[57] Hu B, Jin J, Guo AY, Zhang H, Luo J, Gao G (2015). GSDS 2.0: an upgraded gene feature visualization server. Bioinformatics..

[58] Lee TH, Tang H, Wang X, Paterson AH (2013). PGDD: a database of gene and genome duplication in plants. Nucleic Acids Res.

[59] Tang H, Bowers JE, Wang X, Ming R, Alam M, Paterson AH (2008). Synteny and collinearity in plant genomes. Science..

[60] The Arabidopsis Genome I (2000). Analysis of the genome sequence of the flowering plant *Arabidopsis thaliana*. Nature.

[61] Löytynoja A, Goldman N (2010). webPRANK: a phylogeny-aware multiple sequence aligner with interactive alignment browser. BMC Bioinf.

[62] Wang D, Zhang Y, Zhang Z, Zhu J, Yu J (2010). KaKs_Calculator 2.0: a toolkit incorporating gamma-series methods and sliding window strategies. Genom Proteom Bioinf.

[63] Yang Z, Nielsen R (2000). Estimating synonymous and nonsynonymous substitution rates under realistic evolutionary models. Mol Biol Evol.

[64] Hurst LD (2002). The *Ka/Ks* ratio: diagnosing the form of sequence evolution. Trends Genet.

[65] Krzywinski M, Schein J, Birol I, Connors J, Gascoyne R, Horsman D (2009). Circos: an information aesthetic for comparative genomics. Genome Res.

[66] Kozomara A, Griffiths-Jones S (2014). miRBase: annotating high confidence microRNAs using deep sequencing data. Nucleic Acids Res.

[67] Dai X, Zhao PX (2011). psRNATarget: a plant small RNA target analysis server. Nucleic Acids Res.

[68] Trapnell C, Roberts A, Goff L, Pertea G, Kim D, Kelley DR (2012). Differential gene and transcript expression analysis of RNA-seq experiments with TopHat and cufflinks. Protocols..

[69] Wang L, Cao C, Ma Q, Zeng Q, Wang H, Cheng Z (2014). RNA-seq analyses of multiple meristems of soybean: novel and alternative transcripts, evolutionary and functional implications. BMC Plant Biol.

