# Supplementary material for: Genome-wide identification, phylogeny, and expression analysis of the SBP-box gene family in Euphorbiaceae
Source: BMC Genomics. 2019 Dec 24;20(Suppl 9):912. doi: 10.1186/s12864-019-6319-4 (PMC6929338; doi:10.1186/s12864-019-6319-4)
Supplement: Supplementary file 1 — Additional file 1. This file contains the additional tables (Table S1-S5) associated with the manuscript. Table numbers and titles were listed as follows: Table S1: The information of Euphorbiaceae SBP genes. Table S2: The protein physicochemical properties of Euphorbiaceae SBP proteins. Table S3: The parallel table of scaffold IDs and serial number. Table S4: The information of duplications. Table S5: The identified synteny relationships between Euphorbiaceae species. [file 12864_2019_6319_MOESM1_ESM.docx]

**Table S1.1.** The information of *JcSBP* genes.

| ID | protein | geneID | scaffold | locus | position |
| --- | --- | --- | --- | --- | --- |
| JcSBP1 | XP_020540712.1 | LOC105648779 | 906 | NW_012125302.1 | 3138100..3141000 |
| JcSBP2.1 | XP_012080417.1 | LOC105640657 | 328 | NW_012124487.1 | 3013389..3018163 |
| JcSBP2.2 | XP_012080418.1 | LOC105640657 | 328 | NW_012124487.2 | 3013389..3018164 |
| JcSBP2.3 | XP_020537597.1 | LOC105640657 | 328 | NW_012124487.3 | 3013389..3018165 |
| JcSBP3.1 | XP_020539138.1 | LOC105644836 | 502 | NW_012124748.1 | 3343197..3348341 |
| JcSBP3.2 | XP_012085714.1 | LOC105644836 | 502 | NW_012124748.2 | 3343197..3348342 |
| JcSBP3.3 | xP_020539135.1 | LOC105644836 | 502 | NW_012124748.3 | 3343197..3348343 |
| JcSBP3.4 | XP_020539136.1 | LOC105644836 | 502 | NW_012124748.4 | 3343197..3348344 |
| JcSBP3.5 | XP_020539137.1 | LOC105644836 | 502 | NW_012124748.5 | 3343197..3348345 |
| JcSBP4 | XP_012073540.1 | LOC105635152 | 221 | NW_012124275.1 | 171055..177028 |
| JcSBP5 | XP_012085695.1 | LOC105644822 | 502 | NW_012124748.1 | 3220431..3225206 |
| JcSBP6 | XP_012083646.1 | LOC105643182 | 392 | NW_012124637.1 | 4118388..4121053 |
| JcSBP7 | XP_012091636.1 | LOC105649564 | 684 | NW_012125517.1 | 1720694..1723733 |
| JcSBP8.1 | XP_020540121.1 | LOC105646912 | 905 | NW_012124992.1 | 280362..282830 |
| JcSBP8.2 | XP_020540122.1 | LOC105646912 | 905 | NW_012124992.1 | 280362..282830 |
| JcSBP9 | XP_012082979.1 | LOC105642684 | 392 | NW_012124637.1 | 1332742..1339101 |
| JcSBP10 | XP_012076538.1 | LOC105637624 | 43 | NW_012124081.1 | 413644..415510 |
| JcSBP11 | XP_012069441.1 | LOC105631854 | 119 | NW_012124189.1 | 339983..346770 |
| JcSBP12 | XP_012084189.1 | LOC105643618 | 542 | NW_012124649.1 | 1137690..1145274 |
| JcSBP13 | XP_012067818.2 | LOC105630572 | 46 | NW_012124173.1 | 1110942..1115941 |
| JcSBP14.1 | XP_012087743.1 | LOC105646498 | 897 | NW_012124955.1 | 129784..135564 |
| JcSBP14.2 | XP_012087744.1 | LOC105646498 | 897 | NW_012124955.1 | 129784..135564 |
| JcSBP15 | XP_012083645.1 | LOC105643181 | 392 | NW_012124637.1 | 4111505..4114836 |

**Table S1.2.** The information of *RcSBP* genes.

| ID | protein | gene | scaffold | locus | position |
| --- | --- | --- | --- | --- | --- |
| RcSBP1 | XP_002509450.1 | LOC8258445 | scf_1106159293958 | NW_002994274.1 | 240836..244089 |
| RcSBP2.1 | XP_002510746.1 | LOC8269959 | scf_1106159304512 | NW_002994277.1 | 4528119..4534668 |
| RcSBP2.2 | XP_015575485.1 | LOC8269959 | scf_1106159304512 | NW_002994277.1 | 4528119..4534668 |
| RcSBP2.3 | XP_015575489.1 | LOC8269959 | scf_1106159304512 | NW_002994277.1 | 4528119..4534668 |
| RcSBP2.4 | XP_015575492.1 | LOC8269959 | scf_1106159304512 | NW_002994277.1 | 4528119..4534668 |
| RcSBP3.1 | XP_002511157.1 | LOC8269206 | scf_1106159297664 | NW_002994278.1 | 2218479..2222135 |
| RcSBP3.2 | XP_015578947.1 | LOC8269206 | scf_1106159297664 | NW_002994278.1 | 2218479..2222135 |
| RcSBP3.3 | XP_015578955.1 | LOC8269206 | scf_1106159297664 | NW_002994278.1 | 2218479..2222135 |
| RcSBP4 | XP_002511636.1 | LOC8265831 | scf_1106159290416 | NW_002994279.1 | 359215..361238 |
| RcSBP5 | XP_002514625.1 | LOC8265059 | scf_1106159307312 | NW_002994292.1 | 818966..823715 |
| RcSBP6.1 | XP_002515202.1 | LOC8265895 | scf_1106159298592 | NW_002994294.1 | 886572..892383 |
| RcSBP6.2 | XP_015572376.1 | LOC8265895 | scf_1106159298592 | NW_002994294.1 | 886572..892383 |
| RcSBP7 | XP_002516839.1 | LOC8262359 | scf_1106159301046 | NW_002994316.1 | 428718..435644 |
| RcSBP8 | XP_002517882.1 | LOC8269152 | scf_1106159297894 | NW_002994326.1 | 331682..333827 |
| RcSBP9 | XP_002519316.1 | LOC8261341 | scf_1106159297646 | NW_002994339.1 | 823139..830418 |
| RcSBP10 | XP_002533086.1 | LOC8266790 | scf_1106159292136 | NW_002994966.1 | 67991..69347 |
| RcSBP11.1 | XP_015572162.1 | LOC8286773 | scf_1106159300666 | NW_002994293.1 | 735753..739404 |
| RcSBP11.2 | XP_015572163.1 | LOC8286773 | scf_1106159300666 | NW_002994293.1 | 735753..739404 |
| RcSBP11.3 | XP_015572164.1 | LOC8286773 | scf_1106159300666 | NW_002994293.1 | 735753..739404 |
| RcSBP12.1 | XP_015572189.1 | LOC8286787 | scf_1106159300666 | NW_002994293.1 | 850422..855223 |
| RcSBP12.2 | XP_015572190.1 | LOC8286787 | scf_1106159300666 | NW_002994293.1 | 850422..855223 |
| RcSBP13 | XP_015582800.1 | LOC8277452 | scf_1106159298808 | NW_002994875.1 | 76967..82965 |
| RcSBP14 | XP_015583063.1 | LOC8265833 | scf_1106159290416 | NW_002994279.1 | 367907..370485 |
| RcSBP15.1 | XP_015584550.1 | LOC8264608 | scf_1106159290416 | NW_002994279.1 | 2937478..2942822 |
| RcSBP15.2 | XP_015584551.1 | LOC8264608 | scf_1106159290416 | NW_002994279.1 | 2937478..2942822 |
| RcSBP15.3 | XP_015584552.1 | LOC8264608 | scf_1106159290416 | NW_002994279.1 | 2937478..2942822 |
| RcSBP15.4 | XP_002512054.1 | LOC8264608 | scf_1106159290416 | NW_002994279.1 | 2937478..2942822 |

**Table S1.3.** The information of *HbSBP* genes.

| ID | protein | gene | scaffold | locus | position |
| --- | --- | --- | --- | --- | --- |
| HbSBP1 | XP_021635047.1 | LOC110631502 | 0568 | NW_018746257.1 | 174100..177824 |
| HbSBP2.1 | XP_021635647.1 | LOC110631930 | 0582 | NW_018746271.1 | 731447..740684 |
| HbSBP2.2 | XP_021635648.1 | LOC110631930 | 0582 | NW_018746271.1 | 731447..740684 |
| HbSBP2.3 | XP_021635649.1 | LOC110631930 | 0582 | NW_018746271.1 | 731447..740684 |
| HbSBP2.4 | XP_021635650.1 | LOC110631930 | 0582 | NW_018746271.1 | 731447..740684 |
| HbSBP2.5 | XP_021635651.1 | LOC110631930 | 0582 | NW_018746271.1 | 731447..740684 |
| HbSBP2.6 | XP_021635652.1 | LOC110631930 | 0582 | NW_018746271.1 | 731447..740684 |
| HbSBP2.7 | XP_021635653.1 | LOC110631930 | 0582 | NW_018746271.1 | 731447..740684 |
| HbSBP3.1 | XP_021636923.1 | LOC110632866 | 0620 | NW_018746309.1 | 446046..464494 |
| HbSBP3.2 | XP_021636924.1 | LOC110632866 | 0620 | NW_018746309.1 | 446046..464494 |
| HbSBP4 | XP_021640197.1 | LOC110635250 | 0703 | NW_018746392.1 | 563036..572332 |
| HbSBP5 | XP_021642281.1 | LOC110636760 | 0770 | NW_018746459.1 | 125234..129892 |
| HbSBP6.1 | XP_021642486.1 | LOC110636904 | 0780 | NW_018746469.1 | 191102..193235 |
| HbSBP6.2 | XP_021642487.1 | LOC110636904 | 0780 | NW_018746469.1 | 191102..193235 |
| HbSBP7 | XP_021644278.1 | LOC110638140 | 0824 | NW_018746513.1 | 300697..305903 |
| HbSBP8.1 | XP_021644339.1 | LOC110638184 | 0824 | NW_018746513.1 | 148130..152179 |
| HbSBP8.2 | XP_021644340.1 | LOC110638184 | 0824 | NW_018746513.1 | 148130..152179 |
| HbSBP8.3 | XP_021644341.1 | LOC110638184 | 0824 | NW_018746513.1 | 148130..152179 |
| HbSBP9.1 | XP_021652561.1 | LOC110644195 | 1171 | NW_018746860.1 | 121093..128631 |
| HbSBP9.2 | XP_021652562.1 | LOC110644195 | 1171 | NW_018746860.1 | 121093..128631 |
| HbSBP10 | XP_021658403.1 | LOC110648470 | 1676 | NW_018747365.1 | 52161..56449 |
| HbSBP11.1 | XP_021663003.1 | LOC110651856 | 2990 | NW_018748679.1 | 5614..11743 |
| HbSBP11.2 | XP_021663004.1 | LOC110651856 | 2990 | NW_018748679.1 | 5614..11743 |
| HbSBP11.3 | XP_021663005.1 | LOC110651856 | 2990 | NW_018748679.1 | 5614..11743 |
| HbSBP11.4 | XP_021663006.1 | LOC110651856 | 2990 | NW_018748679.1 | 5614..11743 |
| HbSBP11.5 | XP_021663007.1 | LOC110651856 | 2990 | NW_018748679.1 | 5614..11743 |
| HbSBP11.6 | XP_021663008.1 | LOC110651856 | 2990 | NW_018748679.1 | 5614..11743 |
| HbSBP11.7 | XP_021663009.1 | LOC110651856 | 2990 | NW_018748679.1 | 5614..11743 |
| HbSBP11.8 | XP_021663010.1 | LOC110651856 | 2990 | NW_018748679.1 | 5614..11743 |
| HbSBP12 | XP_021664713.1 | LOC110653404 | 0056 | NW_018745745.1 | 2330809..2337647 |
| HbSBP13.1 | XP_021664715.1 | LOC110653406 | 0056 | NW_018745745.1 | 2316654..2325786 |
| HbSBP13.2 | XP_021664716.1 | LOC110653406 | 0056 | NW_018745745.1 | 2316654..2325786 |
| HbSBP14.1 | XP_021665390.1 | LOC110653897 | 0065 | NW_018745754.1 | 698147..706256 |
| HbSBP14.2 | XP_021665391.1 | LOC110653897 | 0065 | NW_018745754.1 | 698147..706256 |
| HbSBP14.3 | XP_021665392.1 | LOC110653897 | 0065 | NW_018745754.1 | 698147..706256 |
| HbSBP14.4 | XP_021665393.1 | LOC110653897 | 0065 | NW_018745754.1 | 698147..706256 |
| HbSBP15.1 | XP_021667340.1 | LOC110655353 | 0093 | NW_018745782.1 | 1744628..1749492 |
| HbSBP15.2 | XP_021667341.1 | LOC110655353 | 0093 | NW_018745782.1 | 1744628..1749492 |
| HbSBP16.1 | XP_021668748.1 | LOC110656342 | 0105 | NW_018745794.1 | 1841111..1845003 |
| HbSBP16.2 | XP_021668750.1 | LOC110656342 | 0105 | NW_018745794.1 | 1841111..1845003 |
| HbSBP16.3 | XP_021668751.1 | LOC110656342 | 0105 | NW_018745794.1 | 1841111..1845003 |
| HbSBP16.4 | XP_021668752.1 | LOC110656342 | 0105 | NW_018745794.1 | 1841111..1845003 |
| HbSBP17.1 | XP_021674880.1 | LOC110660764 | 0195 | NW_018745884.1 | 928976..960929 |
| HbSBP17.2 | XP_021674882.1 | LOC110660764 | 0195 | NW_018745884.1 | 928976..960929 |
| HbSBP17.3 | XP_021674883.1 | LOC110660764 | 0195 | NW_018745884.1 | 928976..960929 |
| HbSBP18 | XP_021676159.1 | LOC110661735 | 0214 | NW_018745903.1 | 997035..1003960 |
| HbSBP18.2 | XP_021676155.1 | LOC110661735 | 0214 | NW_018745903.1 | 997035..1003960 |
| HbSBP18.3 | XP_021676156.1 | LOC110661735 | 0214 | NW_018745903.1 | 997035..1003960 |
| HbSBP18.4 | XP_021676158.1 | LOC110661735 | 0214 | NW_018745903.1 | 997035..1003960 |
| HbSBP19.1 | XP_021677940.1 | LOC110663044 | 0248 | NW_018745937.1 | 1356737..1359574 |
| HbSBP19.2 | XP_021677941.1 | LOC110663044 | 0248 | NW_018745937.1 | 1356737..1359574 |
| HbSBP20 | XP_021677942.1 | LOC110663045 | 0248 | NW_018745937.1 | 1364423..1366936 |
| HbSBP21 | XP_021681200.1 | LOC110665414 | 0319 | NW_018746008.1 | 749239..766888 |
| HbSBP22 | XP_021682433.1 | LOC110666298 | 0342 | NW_018746031.1 | 1042648..1047232 |
| HbSBP23.1 | XP_021683313.1 | LOC110666939 | 0356 | NW_018746045.1 | 530220..532934 |
| HbSBP23.2 | XP_021683314.1 | LOC110666939 | 0356 | NW_018746045.1 | 530220..532934 |
| HbSBP24 | XP_021683364.1 | LOC110666987 | 0356 | NW_018746045.1 | 542318..551479 |
| HbSBP25 | XP_021686538.1 | LOC110669280 | 0419 | NW_018746108.1 | 812000..818338 |
| HbSBP26.1 | XP_021688870.1 | LOC110670884 | 0458 | NW_018746147.1 | 477301..483977 |
| HbSBP26.2 | XP_021688871.1 | LOC110670884 | 0458 | NW_018746147.1 | 477301..483977 |
| HbSBP26.3 | XP_021688872.1 | LOC110670884 | 0458 | NW_018746147.1 | 477301..483977 |
| HbSBP26.4 | XP_021688873.1 | LOC110670884 | 0458 | NW_018746147.1 | 477301..483977 |
| HbSBP26.5 | XP_021688874.1 | LOC110670884 | 0458 | NW_018746147.1 | 477301..483977 |
| HbSBP26.6 | XP_021688875.1 | LOC110670884 | 0458 | NW_018746147.1 | 477301..483977 |
| HbSBP26.7 | XP_021688876.1 | LOC110670884 | 0458 | NW_018746147.1 | 477301..483977 |

**Table S1.4.** The information of *MeSBP* genes.

| ID | protein | chromosome | position |
| --- | --- | --- | --- |
| MeSBP1 | Manes.01G026000.1.p | LG1 | 4090017..4095735 |
| MeSBP2 | Manes.01G233400.1.p | LG1 | 31583187..31590136 |
| MeSBP3.1 | Manes.01G270700.1.p | LG1 | 34422138..34425071 |
| MeSBP3.2 | Manes.01G270700.2.p | LG1 | 34422138..34425071 |
| MeSBP4 | Manes.03G106900.1.p | LG3 | 18275075..18276403 |
| MeSBP5.1 | Manes.03G189400.1.p | LG3 | 27402871..27410422 |
| MeSBP5.2 | Manes.03G189400.2.p | LG3 | 27402871..27410422 |
| MeSBP6 | Manes.03G211200.1.p | LG3 | 29085006..29091762 |
| MeSBP7 | Manes.05G010400.1.p | LG5 | 683285..690116 |
| MeSBP8 | Manes.05G050800.1.p | LG5 | 3813051..3815281 |
| MeSBP9 | Manes.05G050900.1.p | LG5 | 3821449..3824082 |
| MeSBP10.1 | Manes.05G112200.1.p | LG5 | 10364694..10370035 |
| MeSBP10.2 | Manes.05G112200.4.p | LG5 | 10364694..10370035 |
| MeSBP10.3 | Manes.05G112200.2.p | LG5 | 10364694..10370035 |
| MeSBP10.4 | Manes.05G112200.3.p | LG5 | 10364694..10370035 |
| MeSBP10.5 | Manes.05G112200.5.p | LG5 | 10364694..10370035 |
| MeSBP11 | Manes.05G130100.1.p | LG5 | 15831670..15837893 |
| MeSBP12.1 | Manes.09G032800.1.p | LG9 | 4728670..4738727 |
| MeSBP12.2 | Manes.09G032800.2.p | LG9 | 4728670..4738727 |
| MeSBP13 | Manes.12G009000.1.p | LG12 | 791736..795508 |
| MeSBP14 | Manes.12G010200.1.p | LG12 | 893293..897995 |
| MeSBP15 | Manes.13G009400.1.p | LG13 | 893293..897995 |
| MeSBP16 | Manes.13G011000.1.p | LG13 | 906692..910461 |
| MeSBP17 | Manes.13G144400.1.p | LG13 | 27266607..27270145 |
| MeSBP18 | Manes.14G088200.1.p | LG14 | 7076148..7082993 |
| MeSBP19 | Manes.15G019100.1.p | LG15 | 1543598..1549205 |
| MeSBP20 | Manes.16G029900.1.p | LG16 | 3147390..3149329 |
| MeSBP21 | Manes.17G047500.1.p | LG17 | 18476685..18479800 |

**Table S2.1.** The protein physicochemical properties of middle-sized *SBP* genes.

| Species | Length(aa) | Mw(Da) | Pi |
| --- | --- | --- | --- |
| RcSBP4 | 302 | 33943.34 | 8.92 |
| HbSBP20 | 304 | 34096.64 | 8.96 |
| MeSBP9 | 304 | 33954.4 | 8.96 |
| JcSBP6 | 305 | 34046.39 | 8.96 |
| HbSBP23 | 306 | 34125.52 | 8.9 |
| MeSBP3 | 307 | 34283.98 | 9.02 |
| JcSBP3 | 317 | 34584.44 | 9.15 |
| JcSBP1 | 337 | 37547.6 | 8.77 |
| HbSBP24 | 348 | 38832.32 | 8.7 |
| MeSBP10 | 349 | 38928.16 | 8.98 |
| HbSBP16 | 368 | 41070.02 | 9.44 |
| JcSBP14 | 372 | 39766.78 | 9.13 |
| HbSBP9 | 374 | 39828.04 | 9.13 |
| MeSBP12 | 374 | 39724.86 | 9.31 |
| RcSBP13 | 377 | 40195.24 | 9.38 |
| MeSBP13 | 381 | 41488.26 | 7.99 |
| RcSBP3.1 | 382 | 42091.81 | 8.83 |
| HbSBP10 | 382 | 41653.33 | 8.54 |
| HbSBP8 | 383 | 41551.29 | 8.2 |
| RcSBP11 | 384 | 41919.66 | 8.64 |
| MeSBP15 | 385 | 42137.03 | 8.47 |
| RcSBP14 | 388 | 43342.01 | 8.33 |
| MeSBP8 | 403 | 44573.36 | 7.73 |
| HbSBP19 | 407 | 45351.29 | 7.17 |
| JcSBP15 | 419 | 46714.78 | 7.98 |
| MeSBP16 | 478 | 52743.06 | 8.83 |
| JcSBP5 | 480 | 52818.82 | 8.67 |
| HbSBP7 | 481 | 52656.42 | 8.5 |
| RcSBP12 | 483 | 52963.95 | 8.68 |
| MeSBP14 | 488 | 53231.11 | 8.82 |
| HbSBP11 | 490 | 53195.07 | 8.71 |
| HbSBP15 | 499 | 55011.5 | 6.85 |
| HbSBP5 | 505 | 55820.04 | 7.91 |
| MeSBP1 | 505 | 55813.6 | 7.29 |
| RcSBP15 | 513 | 56430.08 | 7.3 |
| MeSBP18 | 520 | 56274.43 | 6.85 |
| JcSBP2 | 529 | 57483.85 | 8.23 |
| HbSBP14 | 531 | 57105.53 | 7.58 |
| RcSBP5 | 557 | 60183.89 | 8.29 |
| HbSBP17 | 557 | 60075.4 | 5.88 |
| JcSBP13 | 574 | 63487.32 | 7.84 |
| mean value | 418.2439 | 45879.14 | 8.385854 |

**Table S2.2.** The protein physicochemical properties of long-sized *SBP* genes.

| Species | Length(aa) | Mw(Da) | Pi |
| --- | --- | --- | --- |
| HbSBP2 | 764 | 85607.93 | 6.62 |
| MeSBP5 | 770 | 86465.54 | 6.42 |
| MeSBP19 | 774 | 87260.51 | 5.83 |
| JcSBP11 | 780 | 87522.13 | 6.1 |
| RcSBP7 | 795 | 89176.89 | 7.49 |
| HbSBP26 | 813 | 91733.89 | 6.67 |
| MeSBP7 | 978 | 108392.57 | 6.3 |
| MeSBP2 | 982 | 108711.03 | 6.04 |
| JcSBP9 | 983 | 109149.01 | 6.04 |
| HbSBP12 | 1007 | 111575.12 | 6.23 |
| RcSBP6 | 1012 | 111888.79 | 6.38 |
| HbSBP13 | 1012 | 112168.09 | 6.4 |
| HbSBP18 | 1014 | 112053.63 | 5.91 |
| JcSBP12 | 1023 | 113828.26 | 8.42 |
| RcSBP9 | 1026 | 114101.7 | 7.98 |
| MeSBP6 | 1029 | 114083.7 | 8.09 |
| HbSBP4 | 1035 | 114929.75 | 7.64 |
| JcSBP4 | 1068 | 118648.28 | 8.68 |
| RcSBP2 | 1073 | 119319.62 | 8.23 |
| HbSBP25 | 1073 | 118483.85 | 8.72 |
| MeSBP11 | 1074 | 118753.86 | 8.65 |
| mean value | 956.4286 | 106374 | 7.087619 |

**Table S2.3.** The protein physicochemical properties of short-sized *SBP* genes.

| Species | Length(aa) | Mw(Da) | Pi |
| --- | --- | --- | --- |
| HbSBP3 | 140 | 15692.34 | 8.16 |
| MeSBP20 | 140 | 15754.2 | 6.6 |
| MeSBP21 | 140 | 15947.51 | 7.04 |
| RcSBP1 | 141 | 15997.65 | 7.07 |
| JcSBP7 | 142 | 16090.8 | 7.62 |
| JcSBP8 | 194 | 21699.53 | 9.39 |
| HbSBP6 | 195 | 21479.19 | 9.59 |
| HbSBP21 | 195 | 21680.55 | 9.53 |
| RcSBP8 | 198 | 22529.62 | 9.34 |
| RcSBP10 | 198 | 21786.37 | 9.02 |
| MeSBP4 | 202 | 22413.78 | 8.96 |
| MeSBP17 | 203 | 22092.07 | 9.34 |
| JcSBP10 | 211 | 23265.84 | 9.17 |
| HbSBP1 | 213 | 23774.77 | 9.07 |
| HbSBP22 | 219 | 24400.17 | 9.33 |
| mean value | 182.0667 | 20306.96 | 8.615333 |

**Table S3.1.** Scaffold IDs and corresponding locus of physic nut.

| number | scaffold | locus |
| --- | --- | --- |
| scf-1 | 392 | NW_012124637.1 |
| scf-2 | 119 | NW_012124189.1 |
| scf-3 | 684 | NW_012125517.1 |
| scf-4 | 905 | NW_012124992.1 |
| scf-5 | 43 | NW_012124081.1 |
| scf-6 | 221 | NW_012124275.1 |
| scf-7 | 542 | NW_012124649.1 |
| scf-8 | 502 | NW_012124748.1 |
| scf-9 | 897 | NW_012124955.1 |
| scf-10 | 906 | NW_012125302.1 |
| scf-11 | 328 | NW_012124487.1 |
| scf-12 | 46 | NW_012124173.1 |

**Table S3.2.** Scaffold IDs and corresponding locus of castor bean.

| number | scaffold | locus |
| --- | --- | --- |
| scf-1 | scf_1106159290416 | NW_002994279.1 |
| scf-2 | scf_1106159301046 | NW_002994316.1 |
| scf-3 | scf_1106159297894 | NW_002994326.1 |
| scf-4 | scf_1106159292136 | NW_002994966.1 |
| scf-5 | scf_1106159293958 | NW_002994274.1 |
| scf-6 | scf_1106159304512 | NW_002994277.1 |
| scf-7 | scf_1106159297646 | NW_002994339.1 |
| scf-8 | scf_1106159298592 | NW_002994294.1 |
| scf-9 | scf_1106159300666 | NW_002994293.1 |
| scf-10 | scf_1106159298808 | NW_002994875.1 |
| scf-11 | scf_1106159297664 | NW_002994278.1 |

**Table S3.3.** Scaffold IDs and corresponding locus of rubber tree.

| number | scaffold | locus |
| --- | --- | --- |
| scf-1 | 0248 | NW_018745937.1 |
| scf-2 | 0356 | NW_018746045.1 |
| scf-3 | 0582 | NW_018746271.1 |
| scf-4 | 0458 | NW_018746147.1 |
| scf-5 | 0780 | NW_018746469.1 |
| scf-6 | 0319 | NW_018746008.1 |
| scf-7 | 0342 | NW_018746031.1 |
| scf-8 | 0620 | NW_018746309.1 |
| scf-9 | 0568 | NW_018746257.1 |
| scf-10 | 0419 | NW_018746108.1 |
| scf-11 | 0703 | NW_018746392.1 |
| scf-12 | 0214 | NW_018745903.1 |
| scf-13 | 0056 | NW_018745745.1 |
| scf-14 | 2990 | NW_018748679.1 |
| scf-15 | 0824 | NW_018746513.1 |
| scf-16 | 1171 | NW_018746860.1 |
| scf-17 | 0105 | NW_018745794.1 |
| scf-18 | 1676 | NW_018747365.1 |
| scf-19 | 0065 | NW_018745754.1 |
| scf-20 | 0195 | NW_018745884.1 |
| scf-21 | 0770 | NW_018746459.1 |
| scf-22 | 0093 | NW_018745782.1 |

**Table S4.1.** Segmental duplication pairs of four Euphorbiaceae specieses.

| Species | Duplication | Ka | Ks | Ka/Ks | Lacation |
| --- | --- | --- | --- | --- | --- |
| Cassava | MeSBP(1-18) | 0.2313 | 1.5652 | 0.1478 | g10 |
| Cassava | MeSBP(2-6) | 0.2068 | 1.6147 | 0.1281 | g5 |
| Cassava | MeSBP(5-19) | 0.1131 | 0.3599 | 0.3144 | g2 |
| Cassava | MeSBP(7-2) | 0.0617 | 0.3026 | 0.2040 | g5 |
| Cassava | MeSBP(7-6) | 0.1933 | 1.4680 | 0.1317 | g5 |
| Cassava | MeSBP(13-15) | 0.0938 | 0.3200 | 0.2931 | g9 |
| Cassava | MeSBP(17-4) | 0.1478 | 2.0835 | 0.0709 | g3 |
| Cassava | MeSBP(17-21) | 0.1424 | 2.9225 | 0.0487 | g3 |
| Cassava | MeSBP(21-4) | 0.1703 | 1.6216 | 0.1050 | g3 |
| Cassava | MeSBP(21-20) | 0.0790 | 0.3174 | 0.2488 | g3 |
| Cassava | MeSBP(3-9) | 0.0515 | 0.4829 | 0.1066 | g1 |
| Cassava | MeSBP(1-10) | 0.1215 | 0.2438 | 0.4984 | g10 |
| Rubber tree | HbSBP(1-3) | 0.0547 | 0.2873 | 0.1904 | g3 |
| Rubber tree | HbSBP(13-18) | 0.0555 | 0.1898 | 0.2923 | g5 |
| Rubber tree | HbSBP(14-17) | 0.0801 | 0.2161 | 0.3705 | g10 |
| Rubber tree | HbSBP(16-8) | 0.2481 | 1.0604 | 0.2340 | g9 |
| Rubber tree | HbSBP(20-23) | 0.0530 | 0.2484 | 0.2136 | g1 |
| Rubber tree | HbSBP(26-2) | 0.0864 | 0.2939 | 0.2940 | g2 |
| Physic nut | JcSBP(3-1) | 0.1936 | 1.6228 | 0.1193 | g9 |
| Physic nut | JcSBP(8-7) | 0.1394 | 2.9777 | 0.0468 | g3 |
| Physic nut | JcSBP(12-9) | 0.1867 | 1.7028 | 0.1096 | g5 |
| Physic nut | JcSBP(13-2) | 0.2701 | 1.5999 | 0.1688 | g10 |
| Castor bean | RcSBP(1-8) | 0.1650 | 3.1328 | 0.0527 | g3 |
| Castor bean | RcSBP(3-11) | 0.2389 | 2.8132 | 0.0849 | g9 |
| Castor bean | RcSBP(5-15) | 0.2193 | 1.3745 | 0.1595 | g10 |
| Castor bean | RcSBP(6-9) | 0.2071 | 1.3247 | 0.1563 | g5 |

**Table S4.2.** Tandem duplication pairs of four Euphorbiaceae species.

| Species | Duplication | Location |
| --- | --- | --- |
| Manihot | MeSBP(8-9) | g1 and g6 |
| Rubber tree | HbSBP(12-13) | g5 |
| Rubber tree | HbSBP(23-24) | g1 and g6 |
| Rubber tree | HbSBP(19-20) | g1 and g6 |
| Physic nut | JcSBP(6-15) | g1 and g6 |
| Castor bean | RcSBP(4-14) | g1 and g6 |

**Table S5.1.** Synteny *SBP* gene pairs between cassava and physic nut.

| pairs | Group |
| --- | --- |
| MeSBP1-JcSBP2 | g10 |
| MeSBP1-JcSBP13 | g10 |
| MeSBP2-JcSBP9 | g5 |
| MeSBP2-JcSBP12 | g5 |
| MeSBP3-JcSBP6 | g1 |
| MeSBP4-JcSBP7 | g3 |
| MeSBP4-JcSBP8 | g3 |
| MeSBP4-JcSBP10 | g3 |
| MeSBP5-JcSBP11 | g2 |
| MeSBP6-JcSBP9 | g5 |
| MeSBP6-JcSBP12 | g5 |
| MeSBP7-JcSBP9 | g5 |
| MeSBP7-JcSBP12 | g5 |
| MeSBP8-JcSBP15 | g6 |
| MeSBP9-JcSBP6 | g1 |
| MeSBP10-JcSBP13 | g10 |
| MeSBP11-JcSBP4 | g4 |
| MeSBP12-JcSBP14 | g8 |
| MeSBP13-JcSBP1 | g9 |
| MeSBP13-JcSBP3 | g9 |
| MeSBP14-JcSBP5 | g7 |
| MeSBP16-JcSBP5 | g7 |
| MeSBP17-JcSBP7 | g3 |
| MeSBP17-JcSBP8 | g3 |
| MeSBP18-JcSBP2 | g10 |
| MeSBP18-JcSBP13 | g10 |
| MeSBP19-JcSBP11 | g2 |
| MeSBP20-JcSBP7 | g3 |
| MeSBP20-JcSBP8 | g3 |
| MeSBP21-JcSBP7 | g3 |
| MeSBP21-JcSBP8 | g3 |

**Table S5.2.** Synteny *SBP* gene pairs between cassava and castor bean.

| pairs | Group |
| --- | --- |
| MeSBP1-RcSBP5 | g10 |
| MeSBP1-RcSBP15 | g10 |
| MeSBP2-RcSBP6 | g5 |
| MeSBP2-RcSBP9 | g5 |
| MeSBP3-RcSBP4 | g1 |
| MeSBP4-RcSBP1 | g3 |
| MeSBP5-RcSBP7 | g2 |
| MeSBP6-RcSBP6 | g5 |
| MeSBP6-RcSBP9 | g5 |
| MeSBP7-RcSBP6 | g5 |
| MeSBP7-RcSBP9 | g5 |
| MeSBP8-RcSBP14 | g6 |
| MeSBP9-RcSBP4 | g1 |
| MeSBP13-RcSBP3 | g9 |
| MeSBP13-RcSBP11 | g9 |
| MeSBP14-RcSBP12 | g7 |
| MeSBP15-RcSBP3 | g9 |
| MeSBP15-RcSBP11 | g9 |
| MeSBP16-RcSBP12 | g7 |
| MeSBP18-RcSBP5 | g10 |
| MeSBP18-RcSBP15 | g10 |
| MeSBP19-RcSBP7 | g2 |
| MeSBP20-RcSBP1 | g3 |
| MeSBP20-RcSBP8 | g3 |
| MeSBP21-RcSBP1 | g3 |
| MeSBP21-RcSBP8 | g3 |

**Table S5.3.** Synteny *SBP* gene pairs between castorbean and physic nut.

| pairs | Group |
| --- | --- |
| RcSBP1-JcSBP7 | g3 |
| RcSBP1-JcSBP8 | g3 |
| RcSBP2-JcSBP4 | g4 |
| RcSBP3-JcSBP1 | g9 |
| RcSBP3-JcSBP3 | g9 |
| RcSBP4-JcSBP6 | g1 |
| RcSBP5-JcSBP2 | g10 |
| RcSBP5-JcSBP13 | g10 |
| RcSBP6-JcSBP9 | g5 |
| RcSBP6-JcSBP12 | g5 |
| RcSBP7-JcSBP11 | g2 |
| RcSBP8-JcSBP7 | g3 |
| RcSBP8-JcSBP8 | g3 |
| RcSBP9-JcSBP9 | g5 |
| RcSBP9-JcSBP12 | g5 |
| RcSBP11-JcSBP3 | g9 |
| RcSBP12-JcSBP5 | g7 |
| RcSBP15-JcSBP2 | g10 |
| RcSBP15-JcSBP13 | g10 |

**Table S5.4.** Synteny *SBP* gene pairs between cassava and rubber tree.

| pairs | Group |
| --- | --- |
| MeSBP5-HbSBP2 | g2 |
| MeSBP5-HbSBP26 | g2 |
| MeSBP19-HbSBP2 | g2 |
| MeSBP19-HbSBP26 | g2 |
| MeSBP4-HbSBP22 | g3 |
| MeSBP17-HbSBP21 | g3 |
| MeSBP20-HbSBP1 | g3 |
| MeSBP20-HbSBP3 | g3 |
| MeSBP21-HbSBP1 | g3 |
| MeSBP21-HbSBP3 | g3 |
| MeSBP2-HbSBP13 | g5 |
| MeSBP2-HbSBP18 | g5 |
| MeSBP6-HbSBP4 | g5 |
| MeSBP7-HbSBP13 | g5 |
| MeSBP7-HbSBP18 | g5 |
| MeSBP11-HbSBP25 | g4 |
| MeSBP3-HbSBP23 | g1 |
| MeSBP9-HbSBP23 | g1 |
| MeSBP13-HbSBP8 | g9 |
| MeSBP15-HbSBP8 | g9 |
| MeSBP1-HbSBP17 | g10 |
| MeSBP14-HbSBP7 | g7 |
| MeSBP16-HbSBP7 | g7 |
| MeSBP18-HbSBP14 | g10 |
| MeSBP18-HbSBP17 | g10 |

**Table S5.5.** Collinearity *SBP* gene pairs between physic nut and rubber tree.

| pairs | Group |
| --- | --- |
| JcSBP11-HbSBP2 | g2 |
| JcSBP11-HbSBP26 | g2 |
| JcSBP7-HbSBP1 | g3 |
| JcSBP7-HbSBP3 | g3 |
| JcSBP8-HbSBP3 | g3 |
| JcSBP8-HbSBP21 | g3 |
| JcSBP10-HbSBP22 | g3 |
| JcSBP4-HbSBP25 | g4 |
| JcSBP9-HbSBP13 | g5 |
| JcSBP9-HbSBP18 | g5 |
| JcSBP12-HbSBP7 | g5 |
| JcSBP12-HbSBP13 | g5 |
| JcSBP12-HbSBP18 | g5 |
| JcSBP6-HbSBP23 | g1 |
| JcSBP1-HbSBP16 | g9 |
| JcSBP3-HbSBP8 | g9 |
| JcSBP3-HbSBP16 | g9 |
| JcSBP2-HbSBP14 | g10 |
| JcSBP2-HbSBP17 | g10 |
| JcSBP5-HbSBP7 | g7 |
| JcSBP13-HbSBP17 | g10 |

**Table S5.6.** Synteny *SBP* gene pairs between castor bean and rubber tree

| pairs | Group |
| --- | --- |
| RcSBP1-HbSBP1 | g3 |
| RcSBP1-HbSBP2 | g3 |
| RcSBP2-HbSBP25 | g4 |
| RcSBP3-HbSBP16 | g9 |
| RcSBP4-HbSBP23 | g1 |
| RcSBP5-HbSBP14 | g10 |
| RcSBP5-HbSBP17 | g10 |
| RcSBP6-HbSBP13 | g5 |
| RcSBP6-HbSBP18 | g5 |
| RcSBP7-HbSBP2 | g2 |
| RcSBP7-HbSBP26 | g2 |
| RcSBP8-HbSBP3 | g3 |
| RcSBP8-HbSBP21 | g3 |
| RcSBP9-HbSBP4 | g5 |
| RcSBP9-HbSBP13 | g5 |
| RcSBP9-HbSBP18 | g5 |
| RcSBP11-HbSBP8 | g9 |
| RcSBP12-HbSBP7 | g7 |
| RcSBP14-HbSBP19 | g6 |
