# Supplementary figures and images for: Genome-wide identification, phylogeny, and expression analysis of the SBP-box gene family in Euphorbiaceae
Source: BMC Genomics. 2019 Dec 24;20(Suppl 9):912. doi: 10.1186/s12864-019-6319-4 (PMC6929338; doi:10.1186/s12864-019-6319-4)

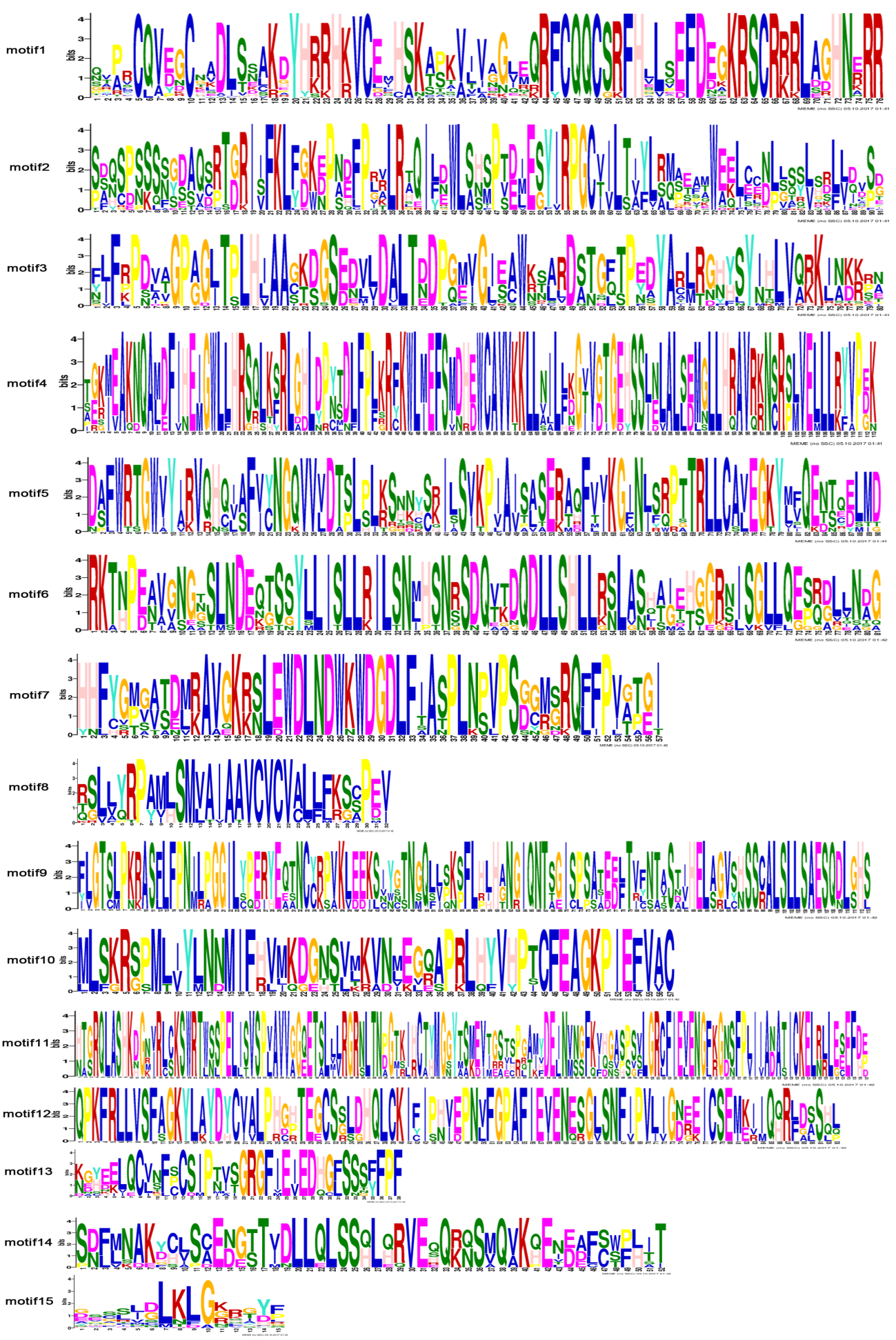


**Figure S1.** The sequence logos of 15 motifs.

Supplement: Supplementary file 2 — Additional file 2: Fig. S1: The sequence logos of 15 motifs. [file 12864_2019_6319_MOESM2_ESM.docx]

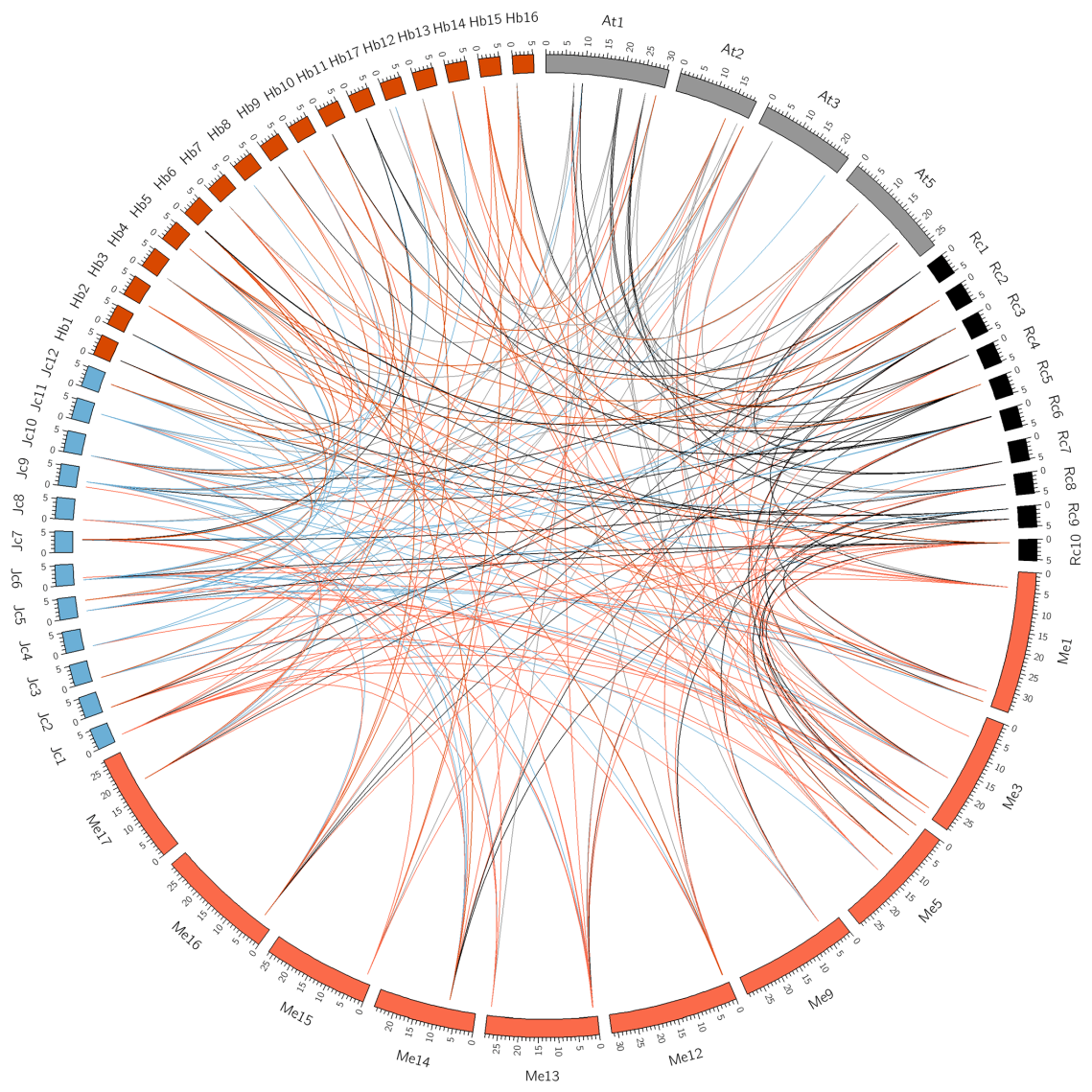


**Figure S2.** The synteny relationships among Euphorbiaceae and *A.thaliana*.

Supplement: Supplementary file 3 — Additional file 3: Fig. S2: The synteny relationships among Euphorbiaceae and A. thaliana. [file 12864_2019_6319_MOESM3_ESM.docx]
